# The Role of Ionic Liquids at the Biological Interfaces in Bioelectronics

**DOI:** 10.1002/advs.202514481

**Published:** 2026-02-08

**Authors:** Yeong‐sinn Ye, Young Jin Jo, Ji Yeon Oh, Ju Yeon Jeong, Lili Guo, Tae‐il Kim

**Affiliations:** ^1^ School of Chemical Engineering Sungkyunkwan University (SKKU) Suwon Republic of Korea

**Keywords:** bioelectronics, biological interfaces, biomimetic engineering, ionic liquids, ion‐transport, neuromorphic systems

## Abstract

The growing demand for personalized healthcare and neurophysiological monitoring is accelerating the advancement of intelligent bioelectronic technologies capable of interacting precisely with biological systems. The human body, as a complex multicellular organism, performs diverse and regulated physiological functions. These biological systems rely on tightly regulated ion‐based mechanisms to respond to stimuli, perceive sensory inputs, and maintain homeostasis. The human nervous system operates as a biologically optimized information processing network with remarkable energy efficiency and adaptability. Efforts to artificially replicate such physiological mechanisms have become a central focus in the development of bioelectronics that establish precise ion‐based interactions with living tissues. Accordingly, this review highlights ionic liquids (ILs) as artificial ionic materials that play a pivotal role in bridging ion‐based signal transmission in biological systems with the electron‐based operation of electronic devices. To realize integrated and multifunctional interfaces capable of engaging with a wide range of biological tissues, a comprehensive understanding of the composition–structure–function relationships and elucidation of the precise working mechanisms of ILs is imperative. Through this, ILs may evolve beyond their traditional role as electrolytes into core platform materials for bioinspired electronic systems that integrate sensing, actuation, and adaptive intelligence.

## Graphical abstract

Ionic liquids (ILs) are highlighted as key artificial ionic materials that bridge biological ion‐based signaling and electronic devices. By understanding their composition, structure, function relationships, and mechanisms, ILs can advance from high performance electrolyte to core materials enabling integrated, multifunctional bioelectronics for precise interaction, sensing, actuation, and adaptive intelligence in diverse biological systems.

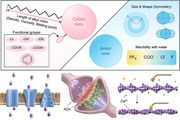

## 1 Introduction

### 1.1 Fundamentals of Bioelectronic Interfaces

**FIGURE 1:**
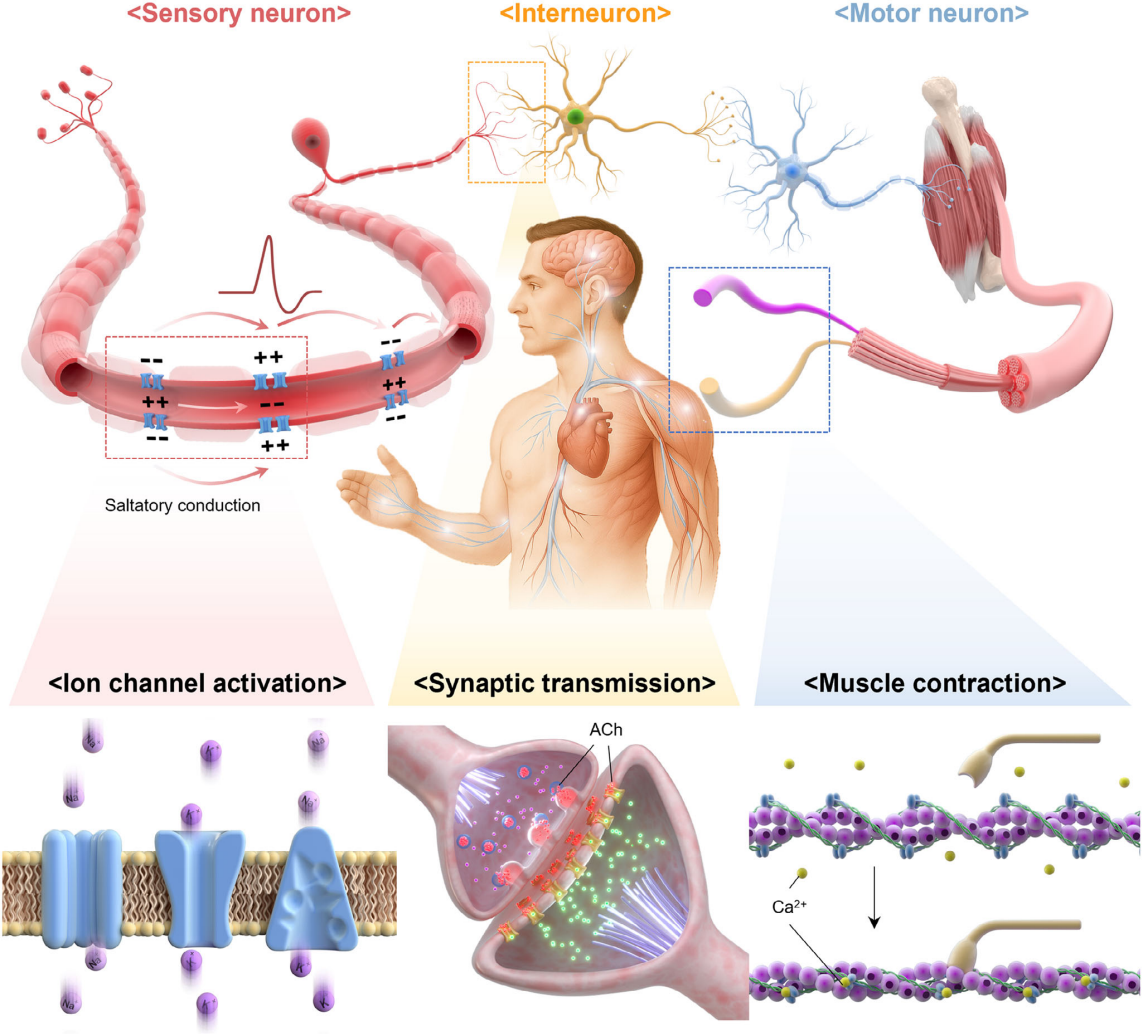
Schematic of ionic interactions within the human biological interface. Surrounding the body, key ionic processes occurring at sensory, inter‐neuronal, and motor synapses are depicted. Ionic movements such as ion diffusion, electrochemical gradients, and membrane depolarization enable the detection of external stimuli, propagation of neural signals through the somatosensory pathway, and subsequent muscle contraction. The schematic illustration highlights how dynamic ionic interactions govern the flow of bioelectrical signals, leading to perception, integration, and motor output in the human nervous system.

Ionic transport and interactions serve as the fundamental basis for physiological signal transmission within the human body. From the detection of external stimuli to the propagation of signals between neurons, and ultimately to muscle contraction, ions act as the primary mediators of such information flows. Upon the detection of external stimuli, sensory receptors activate specific ion channels, initiating selective ion influx or efflux across the cell membrane. This triggers changes in the membrane potential, generating electrical signals that propagate along neurons. Subsequently, synaptic transmission occurs at neuronal junctions, where electrical stimulation leads to the release of neurotransmitters from presynaptic terminals. These neurotransmitters bind to receptors on the postsynaptic membrane, inducing further ionic currents and continuing the signal cascade. Through such repeated ion transport processes, neural signals are rapidly conducted across complex neuronal networks. At the neuromuscular junction, the release of neurotransmitters such as acetylcholine (ACh) activates ion channels on muscle fibers, facilitating a rapid influx of Ca^2+^. This calcium entry initiates intracellular signaling pathways that drive muscle contraction, completing the sensory‐motor loop (Figure 1). Dynamic ionic transfer, including membrane diffusion, electrochemical gradient formation, and membrane depolarization enable precise and integrated signaling across the entire sensorimotor loop, governing complex neural activities such as stimulus perception, information integration, and motor output [1, 2, 3]. The exceptional efficiency and adaptability of biological neural systems stem not only from their structural organization, but also from their localized, reversible, and energy‐efficient ion‐based signal processing [4, 5, 6, 7]. Reproducing these characteristics has been a core objective in the development of bioelectronic interfaces. To effectively interact with biological systems, artificial devices must support ion–electron coupling, ion transport, and electrochemical responsiveness, while also maintaining softness and operational stability in aqueous environments [8].

Ionic liquids (ILs), which are liquid salts composed entirely of ions with organic cations and inorganic/organic anions at room temperature, have recently emerged as highly functional and versatile materials for next‐generation bioelectronic devices that require seamless integration with biological systems [9, 10, 11, 12]. Owing to their high ionic conductivity, wide electrochemical stability window, tunable polarity, and mechanical softness, ILs are ideally suited to mediate signal transmission and amplify signals between electronic systems and biological environments. These materials enable diverse functionalities, including signal transduction, synaptic plasticity, electrochemical gating, and mechanical compliance, making them essential components for the design of precise and biocompatible interfaces [13, 14, 15, 16, 17, 18, 19, 20]. As artificial ionic materials, ILs not only mimic the behavior of biological ions but also actively reshape the physicochemical properties of polymers, transforming conventional bioelectronics into adaptive, bioinspired systems.

This review aims to provide a comprehensive overview of ILs and the IL‐based bioelectronic systems operating through ion‐mediated mechanisms analogous to biological interfaces. In particular, we highlight how ILs contribute to the development of intelligent technologies capable of sensing, stimulation, neuromorphic learning, and closed‐loop control, ultimately enabling more effective interaction at tissue interfaces as a part of bioelectronics. Therefore, we systematically analyze and summarize the functional requirements and suitability of ILs and their chemical functionalities across various application domains, focusing on how the composition design, physicochemical properties, and interfacial interactions with electronic materials influence electrochemical and mechanical performance as well as the operating mechanisms of the devices. Based on these insights, we also emphasize the potential of ILs as multifunctional platform materials in bioelectronics, and discuss perspectives on future bioelectronics for advanced materials and their system‐level integration necessary for their practical realization.

### 1.2 Definition of Ionic Liquids

Ionic liquids represent unique chemical compounds that have garnered significant scientific and industrial interest over the past decades. In general, ILs are defined as salts possessing a melting point at or below 100°C, which distinguishes them from conventional salts molten at high temperature [21, 22, 23]. Most ILs, designated as room‐temperature ionic liquids (RTILs), exist in the liquid state under ambient conditions [24, 25]. The combination of a large, asymmetric organic cation with a diverse range of organic or inorganic anions leads to low melting points of RTILs. Poor packing efficiency and delocalized charge found in ILs prevent crystallization, resulting in lower melting points compared to simple inorganic salts like sodium chloride. It originated by discovering ethylammonium nitrate ([EtNH_3_][NO_3_]), exhibiting a melting point of 12°C, by Paul Walden in 1914 [26]. However, recent research on ILs was promoted by the development of imidazolium‐based ILs in the 1990s, which significantly enabled their various applications [27, 28, 29, 30, 31]. Beyond this simple definition of RTILs, a more fundamental understanding of ILs considers them as highly structured ionic compounds [32, 33, 34]. The constituent cations possess both charged and polar headgroups and nonpolar alkyl chains. These amphiphilic properties lead to self‐assembly to distinguish polar from nonpolar domains, constructing a heterogeneous environment. Such chemical structures, resulting from reciprocal interactions of Coulombic forces, hydrogen bonding, van der Waals interactions, and π–π stacking, give rise to their unique solvation properties and stand out by exhibiting differences from conventional molecular solvents [35, 36, 37, 38, 39]. This unique combination of ionic and molecular characteristics supports their utility in vast applications, from catalysis and electrochemistry to materials science and biotechnology [10, 11, 40, 41, 42, 43, 44, 45, 46]. Consequently, the field has evolved to consider ILs as alternative solvents as well as task‐specific materials, where function can be engineered directly into the molecular structure of the ions.

## 2 Molecular Framework of Ionic Liquids

### 2.1 Classification of Ionic Liquids

**FIGURE 2:**
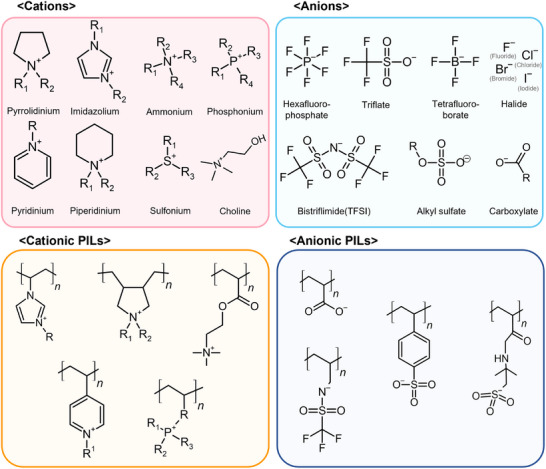
Classification of ionic liquids (ILs). Various cations, anions, cationic PILs (Poly Ionic Liquids), and anionic PILs are illustrated with their names and molecular structures.

Representative cations and anions that are widely used for producing ILs are illustrated in Figure 2. The specific choice of each ion directly dictates fundamental physicochemical characteristics of ILs, including their thermal stability, phase behavior, and ion transport properties (Table 1) [47, 48, 49, 50, 51, 52, 53, 54]. The cation in the form of bulky and asymmetric organic molecules can disrupt the crystalline structures, leading to lower melting points [55, 56, 57]. In general, cations contain various cores such as imidazolium, pyrrolidinium, pyridinium, piperidinium, ammonium, and phosphonium. Among these cations, ILs based on imidazolium and phosphonium are the most commonly used for the development of electrolytes and polymers [58, 59, 60]. Properties of cations can be tuned by the modification of the alkyl substituents (denoted as *R_n_*‐ groups) [61, 62, 63, 64, 65]. The length and branching of these alkyl chains are essential parameters that affect intermolecular interactions and the order of packing density, etc. The longer alkyl chains can introduce stronger van der Waals interactions, resulting in higher viscosity. However, longer and more flexible chains can also induce the structural heterogeneity of the ILs, disturbing efficient ordering of molecules and creating more free volume, which can influence ionic mobility and other transport properties. In addition, the alkyl chain length can determine the application of ILs. For example, in the context of interaction between ILs and lipid bilayers, longer alkyl chains can disrupt the structure of cell membranes more efficiently, as they can induce membrane thinning while shorter molecules only remain in the layer.

**TABLE 1:** Bulky and physical properties of ionic liquids (ILs) with different cations and common anion (Bis(trifluoromethylsulfonyl)imide).

Cation		Abbrev.	Melting point (*T_m_*)(°C)	Density (*ρ*) (g/cm^3^ at 20–25°C)	Viscosity (*η*) (mPa·s at 25°C)	Ionic conductivity (*σ*) (mS/cm at 25°C)	Electrochemical window (V)	Refs.
Imidazolium	1‐Ethyl‐3‐methylimidazolium	EMIM^+^	—17 ∼ —15	1.52	34∼39	6.6∼9.1	4.1∼4.3	[31, 52]
	1‐Butyl‐3‐methylimidazolium	BMIM^+^	—4 ∼ —2	1.44	49∼52	3.4∼3.9	4.1∼4.4	[31, 53]
	1‐Hexyl‐3‐methylimidazolium	HMIM^+^	—9 ∼ —7	1.37	63∼74	1.7∼2.0	4.2∼4.5	[54, 360]
	1‐Octyl‐3‐methylimidazolium	OMIM^+^	—50 ∼ —43	1.28	104	1.1	4.2∼4.5	[47, 360]
Pyrrolidinium	N‐Butyl‐N‐methylpyrrolidinium	BMPyr^+^	—15 ∼ —13	1.40	72∼76	2.2∼2.9	5.5∼5.7	[48, 361, 362]
Piperidinium	N‐Methyl‐N‐propylpiperidinium	PP13^+^	8 ∼ 12	1.41	176	2.1	5.4∼5.7	[49, 363]
Phosphonium	Trihexyl(tetradecyl)phosphonium	P66614^+^	−72	1.07	304	0.4	5.0∼6.0	[50, 51, 364]

While the cations are related to the structure of their ILs, the anions can also determine critical properties of ILs such as their thermal stability, viscosity, and ionic conductivity (Table 2) [66, 67, 68, 69, 70, 71]. The range of available anions includes halides (Cl^−^, Br^−^) and fluorinated organics to inorganic molecules like hexafluorophosphate, tetrafluoroborate, and bis(trifluoromethylsulfonyl)imide, as well as reactive anions such as carboxylates and alkyl sulfates. The thermal stability of ILs relies on the type of anions, as the decomposition temperature can vary by as much as 200°C when the anion is changed for a given cation [72, 73]. Nucleophilicity, basicity, and coordination ability of anions are the main factors in thermal stability. Weakly coordinated anions possess highly delocalized negative charges and form weaker interactions with the cation, resulting in ILs with superior thermal stability. Additionally, a strong negative correlation is related to the hydrophilicity and thermal stability of ILs. More hydrophilic anions tend to produce less stable ILs because of their potential to strongly interact with water, causing increased reactivity, decomposition, and solvation [74, 75]. The anion's structure, such as size, shape, and conformation, also has a dominant effect on transport properties and viscosity of ILs, which is greater than that of the cation [76, 77, 78, 79]. Bulky and flexible anions containing delocalized charge can effectively weaken the electrostatic interactions between cation and anion. This causes reduced intermolecular friction, resulting in lower viscosity, lower glass transition temperatures, and higher ionic conductivity. In contrast, small, symmetric, and highly coordinating anions such as halides can form strong ionic attraction, leading to higher viscosity and lower ionic conductivity. This means that the design of anions is complex and critical in determining various parameters of ILs, considering their applications with specific requirements in solubility for polar compounds, thermal stability, and ionic conductivity.

**TABLE 2:** Bulky and physical properties of ILs with different anions and common cation (1‐Butyl‐3‐methylimidazolium).

Anion	Abbrev.	Melting point (*T_m_*)(°C)	Density (*ρ*) (g/cm^3^ at 20–25°C)	Viscosity (*η*) (mPa·s at 25°C)	Ionic conductivity (*σ*) (mS/cm at 25°C)	Electrochemical window (V)	Refs.
Tetrafluoroborate	BF_4_^−^	−82	1.20	114	4.3	5.5–6.5	[70, 71, 365]
Hexafluorophosphate	PF_6_^−^	10	1.36	312	3.7	4.2–4.5	[69, 365]
Bis(trifluoromethylsulfonyl)imide	Tf_2_N^−^	−4	1.43	52	8.8	4.2–4.4	[31, 68]
Trifluoromethanesulfonate	OTf^−^	16	1.28	90	5.0	4.0–4.2	[31, 67]
Dicyanamide	N(CN)_2_^−^	−21	1.06	34	20.3	3.0–3.5	[66, 366]

Incorporation of the unique functionality of various ILs into macromolecular structures creates polymeric ionic liquids (PILs). In PILs, at least one of the ionic moieties is covalently bonded to or incorporated within polymer chains. These fundamental architectures transform PILs from liquids into solids or gels, opening strong potential for tuning physicochemical properties with the mechanical robustness, processability, and stability of polymers [80, 81]. In general, PILs are constructed through the polymerization of IL monomers. These cations or anions contain polymerizable functional groups, such as vinyl or acrylate moieties [82, 83]. The use of radical polymerization techniques enables precise control of molecular weights, their distribution, and structures, such as in the form of block, graft, or star polymers, which are essential for tailoring various properties of corresponding PILs. As shown at the bottom of Figure 2, PILs are classified based on which ionic moieties are fixed to the polymer chains [84]. Cationic PILs possess cationic moieties such as imidazolium, ammonium, and pyridinium that are covalently bonded to the polymer backbones, either as pendant groups or as parts of the main chain. These PILs also contain free and mobile anions for charge balance. These materials are cores for the development of further applications that utilize the properties of the fixed cation or the interaction with external molecules. On the other hand, anionic PILs consist of polymer chains with carboxylate, sulfonate, or styrenesulfonate functional groups and counter cations. These anionic PILs are less common but are important for specialized applications like single‐ion conductors for next‐generation batteries. The synthesis of cationic versus anionic PILs must be designed by efficient strategies for intended applications. The rational design of the cation–anion pair for ILs and integration with a macromolecular framework for PILs must be conducted for precise tailoring of functions for a wide range of cutting‐edge applications.

### 2.2 Molecular Design and Synthesis of Ionic Liquids

**FIGURE 3:**
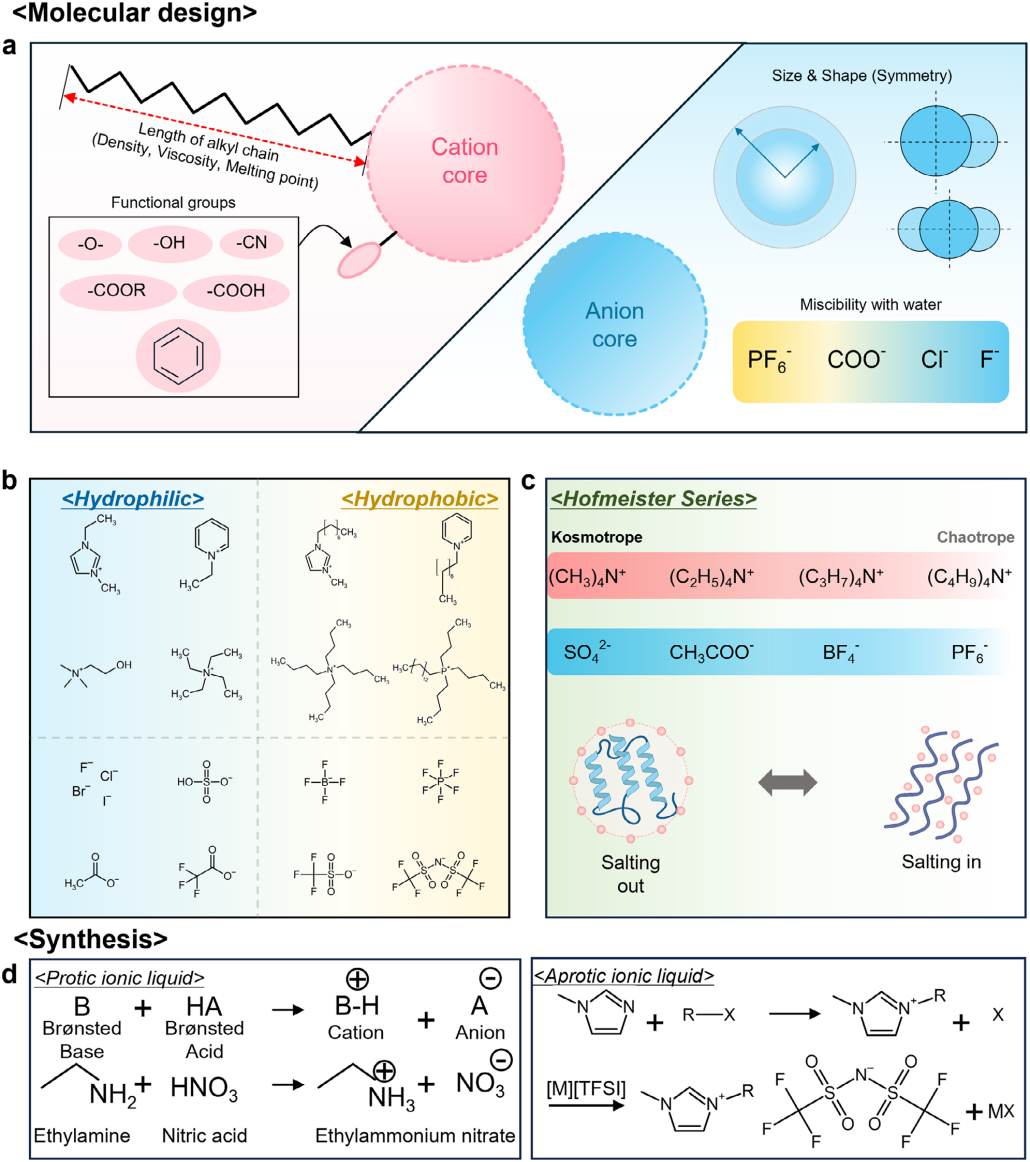
Molecular designs and synthesis of ionic liquids. (a) Parameters in designing cations and anions of ILs and correlated physical and chemical properties. (b) Molecular structures of hydrophilic and hydrophobic cations and anions. (c) Hofmeister series of cations and anions. Kosmotropic ions can be used for “salting‐out” of proteins, and Chaotropic ions used for “salting‐in.” (d) Synthesis of protic and aprotic ionic liquids.

Molecular structures of cations and anions in ILs must be designed by their purpose or tasks, with regard to required characteristics (Figure 3a). The cation is one of the fundamental building blocks of ILs, and its architecture plays a pivotal role in defining physical properties. The most common cores of cations are heterocyclic, such as imidazolium, pyridinium, and pyrrolidinium. Quaternary ammonium or phosphonium cations can also be utilized. The choice of these cores and their subsequent modifications provides powerful opportunities for tuning the IL's characteristics. The most extensively studied modifications of cationic structures are the lengths of the alkyl chains. Numerous works, including formative studies by Huddleston et al., have demonstrated clear and consistent trends [75]. In the case of 1‐alkyl‐3‐methylimidazolium series, which are widely used, longer alkyl chain length generally leads to more viscous and hydrophobic characteristics, while simultaneously decreasing density and surface tension [85, 86]. The increase in viscosity is attributed to stronger van der Waals forces and entanglement between the longer alkyl chains. The decrease in density is a consequence of thin ─CH_2_─ groups replacing the denser and larger imidazolium ring from a volumetric standpoint. In addition, longer chains increase nonpolar character and drive structuration, where the alkyl chains can aggregate to form nonpolar domains, profoundly influencing the IL's solvation and phase separation. Beyond this chain length, the symmetry of cations is another critical factor in determining the melting point [87, 88]. The existence of RTILs is based on the use of asymmetric cations, which can disrupt the formation of stable crystalline structures and suppress the freezing point. On the other hand, utilizing symmetric cations or introducing branched alkyl groups, which can create closely packed structures more efficiently, often results in higher melting temperatures. Moreover, the strategies for the introduction of functional groups onto the cations are the fundamentals for creating ILs, which can serve specific tasks [89, 90]. For example, hydroxyl or ether groups can form hydrogen‐bonding sites, altering solubility and enabling targeted interactions with biopolymers or cellulose [91]. This functionalization transforms the cations from passive structural components into active participants, determining characteristics and functions for their ILs.

While the architecture of cations is important for tuning bulk and physical properties, the anion is widely considered the primary component of chemical functionality, reactivity, and the most defining characteristics of ILs. The choice of anions governs properties such as water miscibility, coordinating ability, and thermal and electrochemical stability [92, 93]. Fundamental distinctions exist between simple, strongly coordinating anions and large, charge‐delocalized, weakly coordinating anions. Such inorganic anions as halides (e.g., Cl^−^ and Br^−^) are small, have a high charge density, and are strong hydrogen‐bond acceptors. ILs containing these anions are typically hydrophilic, highly viscous, and possess relatively low thermal and electrochemical stability. On the contrary, fluorinated anions such as bis(trifluoromethylsulfonyl)imide and hexafluorophosphate (PF_6_^−^) exhibit a highly delocalized negative charge distribution over a large molecular volume. This results in their weak coordination, resulting in ILs that are generally hydrophobic, less viscous, more thermally stable, and possess wider electrochemical windows. This distinction highlights a crucial design principle that the type of anions designates the potential for a specific property, while the cation modulates its magnitude. For instance, the anion determines intrinsic hydrophobicity, but the degree of this hydrophobicity can be tuned by adjusting the alkyl chain length of cations. This hierarchical relationship, forming synergistic effects from cation–anion combinations, is more subtle than simple anionic dominance and is key to reasonable IL design. Furthermore, the anion can be designed as a functional unit to convey specific reactivity. For instance, using a Brønsted basic anion like acetate (OAc^−^) or a hydroxide (OH^−^) creates a basic ionic liquid which is capable of catalyzing reactions like condensations or additions. Xu et al. demonstrated the use of the basic IL ([Bmim]OH) as both the catalyst and the reaction medium for Michael addition reactions, demonstrating how the anion can transform the IL from a passive solvent into an active chemical reagent [94]. This ability to set chemical functions directly into the anion is the basis of specific concepts for ILs.

### 2.3 Hydrophilicity and Hydrophobicity of Ionic Liquid

The interaction of ILs with water is an essential property that governs their application in areas ranging from biocatalysis to biomedical fields. Hydrophilic and hydrophobic cations and anions of representative ILs are illustrated in Figure 3b. The miscibility of ILs with water is primarily indicated by the identity of the anions, a principle firmly established by representative works of Huddleston et al. [75]. The underlying mechanism is the ability of anions to participate in hydrogen bonding [95, 96, 97]. Hydrophilic anions, such as small halides (Cl^−^ and I^−^) and carboxylates (e.g., acetate(COO^−^)), are strong hydrogen‐bond acceptors and interact intimately with water molecules, resulting in complete miscibility. On the other hand, hydrophobic anions, most notably large, fluorinated molecules like bis(trifluoromethylsulfonyl)imide and hexafluorophosphate (PF_6_^−^), are poor hydrogen‐bond acceptors. Their interactions with water are apparently unfavorable compared to the strong hydrogen‐bonding network of bulk water, resulting in phase separation and immiscibility with water. While the anions play dominant roles regarding interaction with water molecules, the cations serve as crucial modulators of hydrophobic properties. Increasing the length of nonpolar alkyl chains on the cation systematically leads to an increase in the overall hydrophobicity of ILs. This effect can be enough to tune the miscibility of ILs. For example, in the 1‐alkyl‐3‐methylimidazolium tetrafluoroborate series, ILs with short alkyl chains (*n* < 6) are miscible with water, however those with longer chains become immiscible [98, 99]. This means that the hydrophobicity of ILs is not an absolute property determined by the kinds of anions alone but arises from the collective contributions of both cations and anions. These results provide a more fundamental understanding of ILs for simply “hydrophilic” or “hydrophobic,” as well as amphiphilic entities. Many ILs, especially those with long alkyl chains or complicated structures, are capable of possessing distinct polar headgroups and nonpolar alkyl or fluorinated tail regions. Research by Koga et al., using a differential thermodynamic probing method, revealed that the TFSI^−^ anion, while forming hydrophobic ILs, concurrently exhibits both the strongest hydrophobic and hydrophilic characteristics among the anions they studied. This inherent amphiphilicity explains the reason why numerous ILs known as hydrophobic can still dissolve substantial amounts of water and tend to form heterogeneous structures with domains separated in polar and nonpolar regions. Therefore, the interaction with water is best described as a tunable balance of amphiphilicity, governed by the relative sizes and characteristics of the hydrophilic and hydrophobic moieties on both cations and the anions.

### 2.4 Hofmeister Series: Salting‐Out and Salting‐In Effects of Ionic Liquids

The Hofmeister series is an observed series of ions based on their consistent ability to affect the solubility of proteins in aqueous solution [100, 101]. The series originally demonstrated the “salting‐out” (precipitation) or “salting‐in” (solubilization) effectiveness of various salts (Figure 3c). Ions at one end of the series, termed kosmotropes (e.g., SO_4_^2−^, HPO_4_^2−^), are strongly hydrated, tend to stabilize protein structures, and promote their precipitation in the solution. On the contrary, ions at the opposite end, termed chaotropes (e.g., SCN^−^, I^−^), are weakly hydrated, leading to destabilization of protein structures and increasing their solubility. Despite their long history and wide applicability in colloid and biological chemistry, the precise molecular mechanism underlying the Hofmeister series remains actively debated. The classical explanation illustrates that the effects are indirect, resulting from the influence of ions on the structure of water from the standpoint of bulk structure and hydrogen bonding. Kosmotropes are considered to enhance the water–water interactions in their vicinity, which prefer the exclusion of solutes such as proteins, leading to aggregation and salting‐out. Meanwhile, chaotropes are known to be intended to disrupt the structures of water molecules, making them more favorable to solvate the solute. However, growing evidence alternatively supports that specific ion effects are dominated by direct interactions between the ions and the solute surface or the air–water interface. These interactions can include electrostatic attraction/repulsion with charged surface groups, dispersion forces between polarizable ions and nonpolar moieties, and competition with water for hydration sites on the solute. For the complicated and structured ions found in ILs, this direct interaction model is more explanatory. Recently, the principles of the Hofmeister series have been extended to ILs to understand and predict their behavior in aqueous systems, particularly in biomedical applications. Several studies have demonstrated that the effects of ILs on protein stability or the mutual solubility of systems consisting of ILs and water which follow Hofmeister‐like trends [102]. This has led to arranging the constituent ions of ILs in the classical Hofmeister framework. Consistent with observations for basic inorganic salts, the anions of the ILs are typically found to apply a more dominant influence on phenomena related to the Hofmeister series than the cations. These are attributed to the higher charge density and stronger hydration of anions compared to the larger organic cations. For example, an early series for several IL ions with stability of ribonuclease A was provided by studies from Constantinescu and Weingärtner [103]. More recent work by Oncsik et al. investigated the aggregation of polystyrene latex particles and allowed for a more detailed placement [104]. These studies, which correlate physicochemical effects with identities of ions, are crucial for constructing predictive frameworks for behaviors of ILs in complex aqueous environments.

### 2.5 Synthesis of Ionic Liquids

Here, two main synthetic processes for ILs are summarized in Figure 3d [105, 106]. Synthesis of protic ILs is accomplished by formation through proton transfer in a Brønsted acid–base neutralization reaction. On the other hand, that of aprotic ILs is based on formation through covalent bond formation (quaternization) followed by anion exchange (metathesis). The main difference in synthesis between protic and aprotic ILs lies in the nature of the chemical bonds of the cations. Protic ILs can be produced via coordinated covalent bonds, known as proton transfer (B+HA→B‐H^+^+A^−^). In contrast, the cations of aprotic ILs are created through an alkylation reaction (R‐X+Imidazole→R‐Imidazole^+^+X^−^), which is a standard covalent bond formation. The main challenge in this pathway is the removal of byproducts generated in the subsequent anion exchange step. This distinction in synthetic pathways between protic and aprotic ILs is the key to understanding why the two types of ILs exhibit cost, purity issues, and unique properties.

Even if this approach has been widely used, the conventional synthetic pathway for ILs is still congested with significant challenges, the most critical of which is product purity. The anion metathesis step, particularly when starting from halide salts, is prone to leaving residual halides as impurities in the product [107, 108, 109]. Even tiny amounts of halides (e.g., Cl^−^, Br^−^) can have a dramatic and detrimental effect on the physicochemical properties of the ILs. These impurities can significantly change viscosity, density, phase behavior, and electrochemical stability, and can serve as poisons in biological applications. Producing pure ILs is a major and vital issue in this field, as it results in inconsistencies in reported data and a lack of reproducibility between labs or even between different commercial batches of the same ILs. This means that the selected synthesis is not merely a practical detail but a hidden variable that fundamentally defines the properties of the final ILs. ILs synthesized via a halide‐based metathesis are not truly the same substances as those prepared via a halide‐free route. Beyond the purity, the conventional synthesis routes also face sustainability challenges [110]. Several requirements, such as the large amounts of organic solvents for reactions or extractions, the significant salt waste from metathesis, and the heavy dependence on expensive and stoichiometric reagents like silver salts, all contribute to a poor economy and a substantial environmental footprint. These increase the cost and complexity of production while creating a fundamental trade‐off between cost, purity, and performance of ILs. The cheapest and most common synthetic routes are closely related to the most impure products, while high‐purity methods are naturally more expensive. This relationship remains a hurdle to ILs being adopted as a component in industries. In response to the issues of conventional synthesis, numerous efforts have been made toward greener and more sustainable protocols for producing ILs. Primary focuses have been the development of halide‐free synthesis that avoids the problematic metathesis steps, thus removing the source of halide contamination. For example, as reported by Stagel et al. (2022), it is a two‐step and one‐pot procedure for synthesizing TFSI‐based ILs [111]. In this method, an alkyl bistriflimide was first prepared and then used directly as the alkylating agent to quaternize an amine or phosphine. This reaction, accompanied by high atom efficiency, can produce no salt byproducts and intrinsically halide‐free ILs with simple purification. In addition, an emerging trend is the development of bio‐based ionic liquids derived from renewable resources. This approach aims to reduce the reliance on petrochemical precursors and create ILs with improved biodegradability and lower toxicity. ILs are synthesized using carbohydrates, amino acids, and other natural products. Ferlin et al. have explored carbohydrate‐derived ILs, while others have shown that ILs based on amino acid esters can display remarkably lower cytotoxicity than conventional ILs [112]. These approaches contribute to more important discussions of the green ILs. The assessment from the standpoint of eco‐friendliness is essential, considering the energy consumption and toxicity of the synthesis, the efficiency of recycling, and the biodegradability. Green chemistry should contain inherently green molecules as well as the design of greener processes where ILs are used as key components of a sustainable system. This paradigm suggests designing a green molecule and engineering a sustainable system to resolve complex and realistic challenges.

## 3 Functional Roles of Ionic Liquids at Biointerfaces

### 3.1 Electrochemical Properties of Ionic Liquids

**FIGURE 4:**
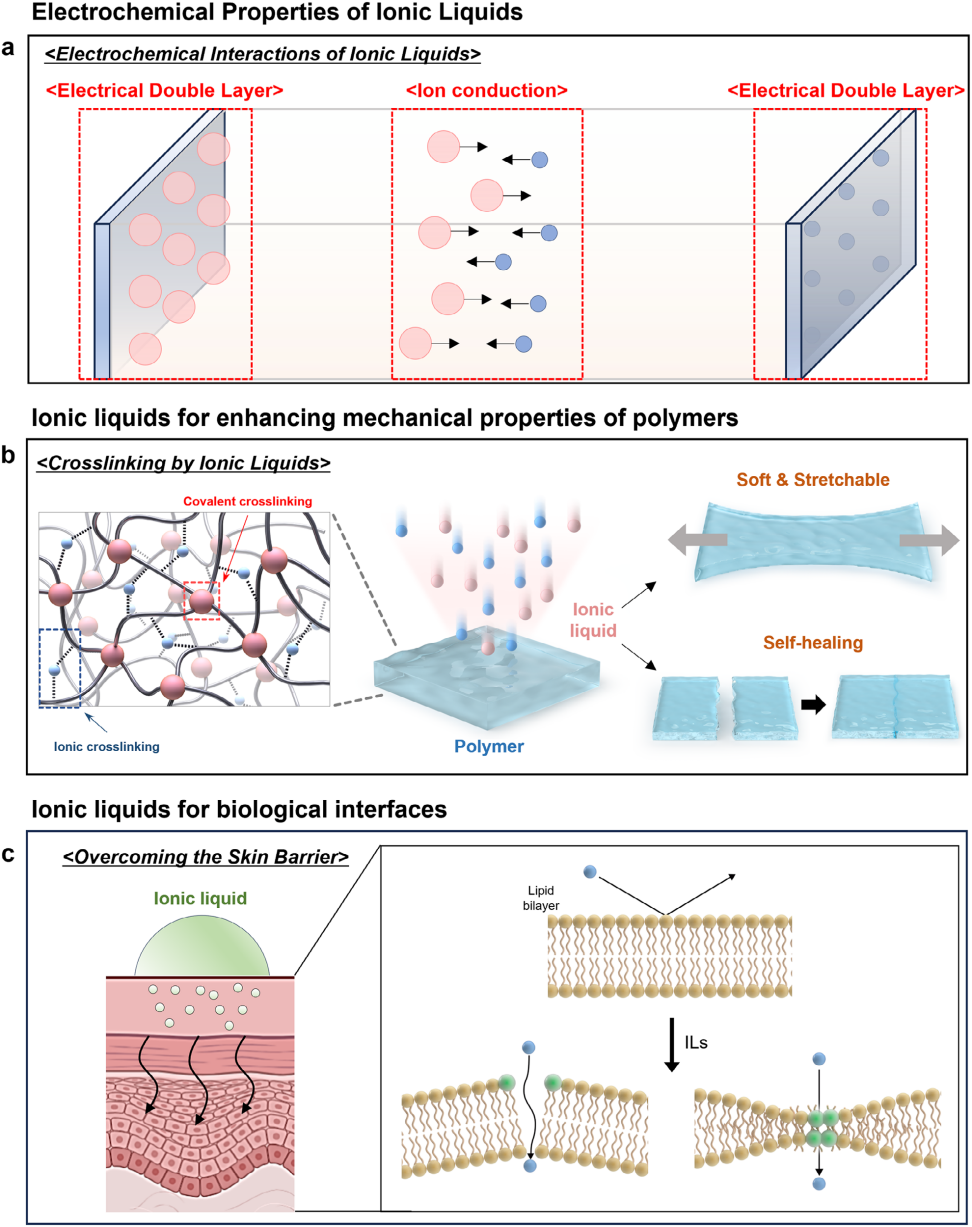
Ionic liquid‐tunable properties for biointerfaces. (a) Electrochemical properties of ionic liquids, demonstrating “Electrical Double Layers” (EDLs) and ion conduction between two metal electrodes. (b) Enhancing the mechanical properties of polymers by inducing ionic liquids, which can form ionic and covalent crosslinking, leading to softness, stretchability, and self‐healing characteristics. (c) Ionic liquids can be used for more efficient delivery of molecules or drugs by disrupting the structure of lipid bilayers.

This part demonstrates ion conduction and the electrical double layer (EDL) formed by ILs in electrolytic systems (Figure 4a). This corresponds to the fundamental electrochemical properties that enable a variety of applications, such as electronic devices or biosensors. Compared to conventional electrolytes, which consist of salts and solvents, molecules of ILs are conductive themselves as they have ionic charges [113, 114, 115, 116]. Therefore, ion conduction occurs through the movement of solvated ions as well as the motion of ions. The size, shape, and viscosity of ions and the interactions between them determine the ionic conductivity of ILs. The conductivity of RTILs, for example, imidazolium‐based ILs, has a range from 1–10 mS/cm, such as 1‐ethyl‐3‐methylimidazolium dicyanamide, exhibiting outstanding ionic conductivity of up to 27 mS/cm [66, 117]. This intrinsic ionic conductivity, together with extremely low vapor pressure and thermal and chemical stability, makes ILs ideal ionic conductors. At the interfaces between electrodes and electrolytes, ILs can form the EDL, which is different from that of conventional electrolytes. While the EDL of traditional electrolytes is analyzed by diffused layers and the Gouy–Chapman model, the EDL of ILs is studied as forming highly ordered structures of alternating cations and anions in several nanometers thickness from the surface of electrodes [118, 119, 120, 121]. These structures can screen the charge on the electrode surface more efficiently over a short distance, which is attributed to extremely high capacitance. These properties are core operating principles for supercapacitors and electrolyte‐gated transistors with outstanding performance, even when driven by low voltages.

### 3.2 Ionic Liquids for Enhancing Mechanical Properties of Polymers

This section is demonstrated to explore how ILs can create softer, more stretchable, and self‐healing materials when introduced into a polymer matrix (Figure 4b). ILs can serve as additives as well as active components that can tune the structure and properties of polymers. The underlying mechanisms are ionic crosslinking, covalent crosslinking, and plasticization [122, 123, 124]. By integrating ionically charged (positively or negatively charged) functional groups into polymer chains, the cations and anions of the ILs can form reversible ionic bonding with these groups through electrostatic attraction. These interactions can form 3D networks, lending mechanical strength and stability to the polymers. Reversible ionic crosslinking is one of the fundamental principles demonstrating self‐healing properties of polymers [125, 126, 127, 128]. When damaged areas are contacted, the ILs facilitate the rearrangement of ions, allowing fresh ionic bonds to form across the interface, thereby recovering mechanical properties. ILs are effective plasticizers for biopolymers such as starch, chitosan, and cellulose. These biopolymers are naturally hard and brittle due to exceptional hydrogen bonding between their polymeric chains. ILs can penetrate between these polymeric chains and disrupt these hydrogen bonds more effectively. This process dramatically increases the mobility of the polymeric chains, extensively improving flexibility and stretchability. For example, the addition of 1‐butyl‐3‐methylimidazolium chloride to starch films demonstrates significant enhancement of tensile strength and elongation at break from 1.38 ± 0.45 to 8.93 ± 1.59 MPa and from 8.06 ± 0.05 to 29.53 ± 0.86%, respectively [129]. The dynamic nature of ionic crosslinking allows autonomous self‐healing of the material after damage without additional treatments. When cut surfaces are contacted together, ILs facilitate the movement of polymer chains and ions, allowing electrostatic crosslinks to recover damaged interfaces and mechanical properties. A representative example of a self‐healing polymer electrolyte contains 1‐ethyl‐3‐methylimidazolium bis(trifluoromethylsulfonyl)imide ([EMIM][TFSI]), exhibiting its recovery of over 80% of its initial tensile strength after being cut and healed. In detail, the initial breaking strength of 45 kPa and an elongation at break of 400% were recovered after self‐healing to a breaking strength of 35 kPa and an elongation of 340% [130]. Furthermore, a polymeric ionic liquid (PIL) hydrogel exhibited outstanding self‐healing efficiency of 85% with a post‐healing tensile strength reaching 2.5–3.9 MPa, compared to 5.8MPa of the hydrogels before being cut [131]. By utilizing these mechanisms, various functional materials that overcome the limitations of conventional materials can be fabricated. Introducing an ionic liquid within a polymer matrix can make gel polymer electrolytes (GPEs) and ionogels. It is possible to create solid‐state materials that possess improved mechanical properties while maintaining high ionic conductivity. These materials are key components for flexible and stretchable energy storage devices (batteries and supercapacitors) and wearable electronics.

### 3.3 Ionic Liquids for Drug Delivery at the Biointerfaces

The interaction between ILs and the lipid bilayer of cell membranes is demonstrated in Figure 4c. These schematic illustrations are used to explain drug delivery, antimicrobial action, and the concept of cytotoxicity. Activities of ILs at diverse biointerfaces are derived from their ability to disrupt the structures of lipid bilayers, known as the fundamental components of cell membranes [132, 133, 134]. The key to this mechanism is the amphiphilic characteristic of cations. These cations possess both charged, hydrophilic heads and hydrophobic alkyl chains of tails. The interaction proceeds through the following steps [135, 136, 137, 138]:

1. Insertion: The hydrophobic tails of the IL cations penetrate into the hydrophobic parts of the lipid bilayers.
2. Swelling and Disorganization: This insertion disrupts the highly ordered structure of the lipid tails, leading to membrane swelling and a more fluid, disorganized state. ILs decrease the thickness of the lipid bilayer, and their cations accumulate between the lipid head and tail regions.
3. Permeabilization: Penetration of ILs into lipid bilayers makes them more permeable, allowing external molecules like drugs or dyes to move through more easily.
4. Disintegration: ILs at high concentrations can serve as surfactants, disintegrating the lipid bilayer and leading to cell lysis.

The degree of these disruptions is strongly connected to the hydrophobic properties of ILs. The longer alkyl chains on the cations increase the hydrophobic characteristics, resulting in deeper insertion into the lipid bilayer and the production of more severe disruption and biological effect (enhancement in drug permeation, antimicrobial activity, and cytotoxicity) [139, 140, 141]. The mechanism of lipid bilayer disruption is effective in Transdermal Drug Delivery Systems (TDDS). The biggest obstacle to transdermal drug delivery is the stratum corneum, the outermost layer of the skin. This layer consists of highly ordered lipid structures, which can perfectly block the penetration of foreign substances. ILs can reversibly disrupt these lipid structures through the mechanism described above, opening pathways for drugs or molecules [142, 143, 144]. However, given that drug delivery has been thoroughly addressed in prior studies, we will limit our discussion of the finer details in the present work [134, 145, 146].

Thus, IL‐induced membrane disruption is highly structure‐dependent. Hydrophobic cations with long alkyl chains exhibit stronger insertion and are therefore associated with higher cytotoxicity [147]. In contrast, hydrophilic and short‐chain ILs, particularly those based on choline, ammonium, or amino acids, tend to interact more weakly with lipid bilayers and are generally considered suitable for use at tissue or skin interfaces [133, 141]. These biocompatible ILs disrupt lipid packing only mildly and in a reversible manner, which allows functional ion transport without causing irreversible membrane damage (Table 3).

## 4 Ionic Liquid‐Integrated Advanced Applications

### 4.1 Bioelectronic Interfaces for Sensory and Motor

#### 4.1.1 Sensory Input Interface: Sensors and Transistors

The interface between electronic materials and biological systems is critically governed by the ability to transduce the pervasive ionic activity inherent to living organisms into precise and reliable electrical signals. The human body continuously generates diverse biosignals in response to external environmental stimuli and for the maintenance of internal homeostasis, and these are primarily expressed as electrical signals through ion transport and changes in membrane potential. Such mechanisms of biosignal transduction closely mirror the operating principles of sensors and transistors. Sensors respond electrically through changes in resistance or current to external stimuli, while transistors operate by modulating current flow via doping and dedoping processes induced by ions injected through the gate. This ion‐mediated signal detection and amplification architecture serves as a fundamental bridge between biological systems and electronic devices, thereby necessitating the integration of materials optimized for this unique interface [148, 149].

In this context, ILs have emerged as highly promising interface materials for sensors and transistors. While metal electrodes such as Ag/AgCl or carbon‐based conductors provide excellent electronic conductivity, they inherently lack ionic transport capability, leading to high interfacial impedance and limited coupling to biological ionic flux. Metal electrodes typically rely on liquid electrolytes to reduce impedance, but these electrolytes dry within a few hours upon air exposure, degrading signal quality and limiting long‐term electrophysiological monitoring. Although additional strategies, such as conformal attachment, can reduce contact impedance, dry electrodes often suffer from motion‐induced artifacts [150, 151]. By contrast, the high capacitive coupling, mechanical compliance, and chemical stability of ILs minimize such motion artifacts and support stable, low‐voltage modulation of mixed ionic–electronic channels [152]. In addition, coating rigid metal or conducting polymer electrodes with IL‐based gels forms a soft, conformal ionic interface with high ionic conductivity and large interfacial capacitance. This configuration substantially lowers skin electrode impedance and enables stable signal recording over periods ranging from hours to multiple days [153]. Their inherently high ionic conductivity enables fast and precise transduction of subtle biological ionic signals. Moreover, ILs support the fabrication of mechanically flexible devices through uniform dispersion within polymer matrices and contribute to enhanced doping and dedoping efficiency, thereby improving signal amplification and response speed in transistors. Beyond functioning as conventional electrolytes, ILs actively modulate electronic signals via capacitive gating, channel charge density regulation, and dielectric response tuning. These multifunctional capabilities allow ILs to play a central role in signal detection, amplification, and modulation at the electronic–biological interface. As a result, ILs have proven particularly effective in ion–electron coupling devices such as organic electrochemical transistors (OECTs), organic field‐effect transistors (OFETs), and electrolyte‐gated transistors (EGTs) [154, 155, 156, 157].

**FIGURE 5:**
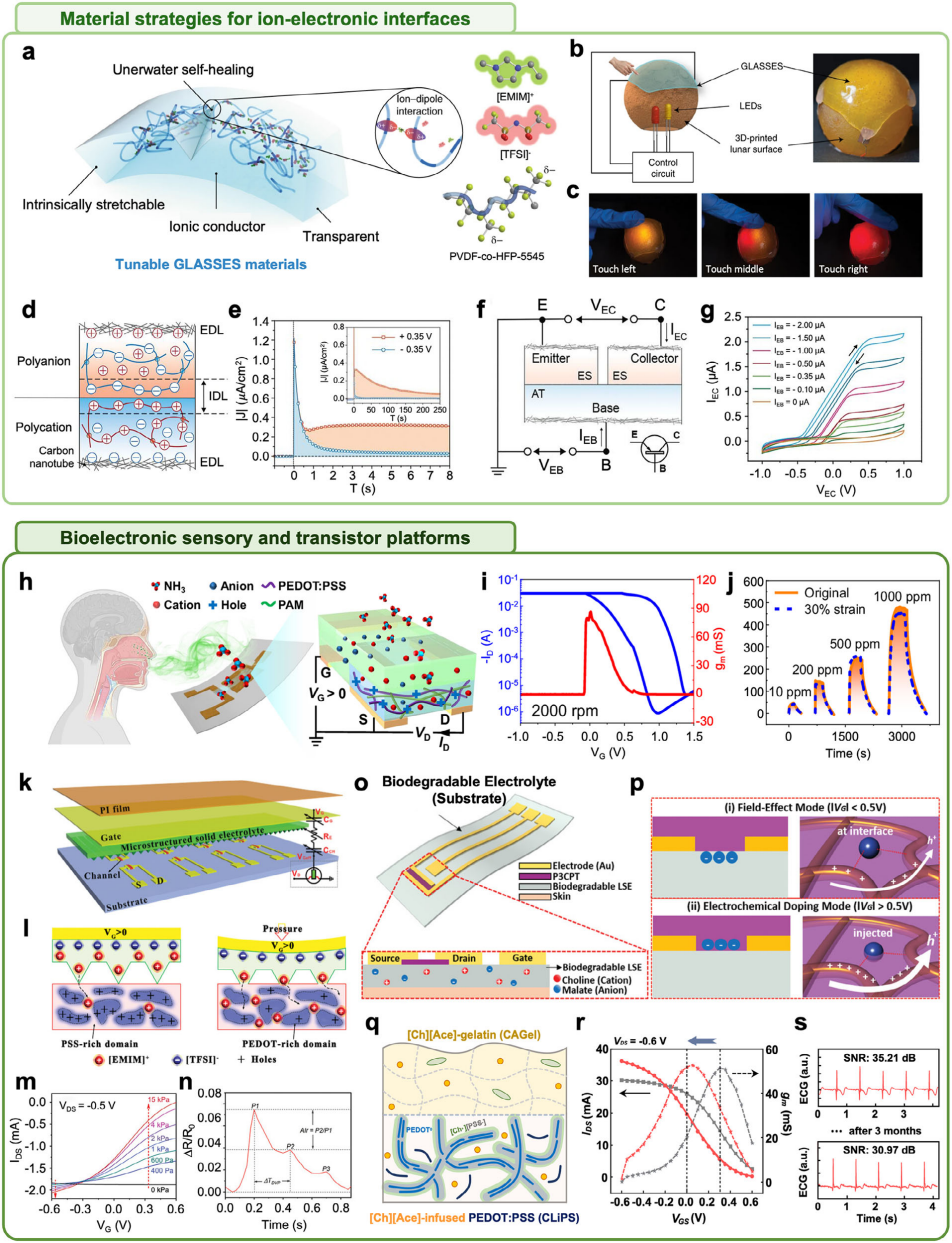
Role of ions in sensory interface: sensors and transistors based on ionic liquids. (a) Schematic of GLASSES self‐healing via reversible ion–dipole interactions in P(VDF‐HFP)/[EMIM][TFSI]. Hydrophobic TFSI^−^ enables healing even under wet conditions. (b) A conformable pressure sensor with GLASSES film conforming to 3D lunar surfaces, demonstrating flexibility and adaptability. (c) Pressure modulated LED illumination through changes in interfacial capacitance, illustrating IL‐based mechanical to electrical transduction. Reproduced with permission [160]. Copyright 2019, Springer Nature. (d) ES/AT heterojunction with CNT electrodes forming low impedance electric double layers for capacitive coupling without redox reactions. (e) Ionic current response under ±0.35 V bias reveals strong rectification behavior due to ionic double layer (IDL) collapse and ion drift in forward bias. (f) Trilayer ionoelastomer transistor (ES/AT/ES) operating via capacitive ionic switching in a soft, stretchable, and gel‐free structure. (g) Output characteristics show drain current modulation (I_EC_) by input current (I_EB_), confirming ionic transistor behavior based on ion doping/dedoping. Reproduced with permission [162]. Copyright 2020, The American Association of the Advancement of Science. (h) NH_3_ gas sensing via ion‐analyte interaction in all‐gel OECT, where polar NH_3_ donors reduce PEDOT doping level via interaction with [THMA][MeSO_4_], lowering hole conductivity. (i) Transfer characteristics of the OECT with a 1.8 µm gel layer show high transconductance (86.4 mS), indicating efficient ion penetration and charge transport. (j) NH_3_ response maintained after 30% strain, confirming mechanical durability and functional reliability for stretchable gas sensing. Reproduced with permission [171]. Copyright 2025, Springer Nature. (k) Schematic of pressure‐sensitive OECT design with pyramid microstructure utilizing a [EMIM][TFSI]‐PVDF‐HFP ionogel. (l) Schematic of the ion interaction mechanism via pressure‐induced contact area modulation. (m) Device output demonstrates pressure‐sensitive ion doping, where increased pressure enhances gate–electrolyte capacitance and source–drain current. (n) Sensor detects real‐time radial artery pulses, highlighting the capability for wearable biosignal monitoring. Reproduced with permission [173]. Copyright 2020, John Wiley and Sons. (o) Structure of biodegradable levan/[Ch][MA] transistor, enabling substrate–dielectric integration for direct skin or tissue contact. (p) Dual mode transistor operation: (top) field‐effect modulation at low V_GS_ and (bottom) electrochemical doping at higher V_GS_. Reproduced with permission [15]. Copyright 2020, John Wiley and Sons. (q) Schematic of channel modification using a [Ch][Ace] ionic liquid and an ionic gel electrolyte (CAGel). (r) Transfer and transconductance characteristics of OECTs comparing pristine and IL‐modified channels. (s) Long‐term stability of [Ch][Ace] IL‐based OECTs is demonstrated by stable ECG recordings over extended operation periods. Reproduced with permission [157]. Copyright 2025, John Wiley and Sons.

A representative example of IL‐enabled interface design is reported by Yue Cao et al., wherein [EMIM][TFSI], an IL containing the hydrophobic anion TFSI^−^, was integrated into an amorphous elastomer matrix of poly(vinylidene fluoride‐co‐hexafluoropropylene) (P(VDF‐HFP)) to fabricate a self‐healing electronic skin named GLASSES (Figure 5a) [158, 159, 160]. The delocalized charge distribution and steric hindrance of TFSI^−^ suppress hydrogen bonding, minimize water uptake, and ensure stable operation under humid or submerged environments. This IL‐polymer composite exhibited enhanced ionic conductivity and mechanical compliance, enabling its application on hemispherical 3D surfaces (Figure 5b). Upon mechanical pressure, local capacitance changes at the skin‐sensor interface modulated the brightness of embedded LEDs, demonstrating reliable pressure‐sensing behavior (Figure 5c) [161]. In this system, the IL served not only to reinforce mechanical flexibility and ionic conductivity but also to act as a dynamic transduction medium that converted mechanical deformation into electrical outputs.

The functionality of ionic liquids (ILs) has been further extended by integrating them into ionically crosslinked polymer networks, resulting in solid‐state ionoelastomers capable of exhibiting diode and transistor‐like electronic behaviors [162]. In these systems, polymer chains bearing fixed anionic or cationic groups restrict the mobility of counterions, thereby generating asymmetric ionic transport pathways (Figure 5d) [163]. For instance, 1‐ethyl‐3‐methyl imidazolium (3‐sulfopropyl) acrylate (ES) contains immobilized anionic moieties (polyanion) and mobile cations, whereas 1‐[2‐acryloyloxyethyl]‐3‐butylimidazolium bis(trifluoromethane) sulfonimide (AT) incorporates immobilized cationic moieties (polycation) and allows free anion migration [164, 165]. When arranged in an ES/AT heterojunction, the interface promotes entropy‐driven rearrangement of mobile ions, leading to the formation of an internal electrostatic field. This field gives rise to drift currents that counterbalance diffusion currents, thereby enabling rectification through purely ionic redistribution mechanisms. Such ionic rectification behavior can be theoretically explained by the asymmetric current response under positive and negative bias voltages. Under a forward bias of +0.35 V, the current exhibits a slow exponential decay with a relaxation time (τ) of 71.0 ± 0.3 s, whereas under a reverse bias of −0.35 V, a much faster decay is observed with 0.32 ± 0.01 s (Figure 5e). The total accumulated charge densities, obtained by integrating the current curves, are 33 ± 5 mC/cm^2^ for the forward bias and 0.7 ± 0.1 mC/cm^2^ for the reverse bias, clearly indicating rectification behavior. This marked difference in decay rates and charge accumulation theoretically supports that effective rectification can be achieved purely through ionic rearrangement at the interface, without electronic conduction. Furthermore, a trilayer ES/AT/ES structure demonstrates a bipolar ionic transistor behavior, wherein the outer ES layers act as emitter and collector, and the central AT layer functions as the base (Figure 5f) [162]. Voltage sweep measurements between −1 and 1 V confirm input–output modulation characteristics based solely on doping/dedoping dynamics of ionic carriers (Figure 5g). These findings offer compelling evidence for the realization of fully ionic transistor devices with no reliance on electronic charge carriers, marking a significant advancement in soft ionotronic systems.

Building on these material‐level innovations, subsequent research has explored the integration of ILs as active components in bioelectronic devices, particularly in OECTs, to achieve multimodal biosignal detection [12, 166, 167]. These systems have been engineered to sensitively transduce a broad range of biological signals, including chemical, mechanical, and electrophysiological signals, with high fidelity and structural adaptability [168, 169, 170]. Among these, certain OECT platforms have been tailored to target chemical signaling associated with olfactory sensing, where ILs such as 1‐vinyl‐3‐butylimidazolium tetrafluoroborate ([VBIm][BF_4_]) were polymerized within a poly(acrylamide)‐based poly(3,4‐ethylenedioxythiophene):poly(styrene sulfonate) (PEDOT:PSS) double‐network gel [171]. This all‐gel configuration simultaneously enabled high ionic conductivity and mechanical flexibility, while eliminating the need for external liquid electrolytes (Figure 5h). The device achieved a peak transconductance of 86.4 mS with an active layer thickness of 1.8 µm (Figure 5i). Notably, the IL served a functional role in gas sensing by directly interacting with polar gaseous analytes such as ammonia (NH_3_). Acting as an electron donor, NH_3_ suppressed the doping state of PEDOT, reducing hole carrier density and thereby decreasing the drain current [172]. This ion‐analyte interaction‐driven signal modulation mechanism demonstrates the potential of such devices for bioinspired olfactory sensing applications. Furthermore, the device retained stable sensing performance under 30% perpendicular tensile strain, underscoring the excellent mechanical elasticity and stretchability of the IL‐based platform (Figure 5j) [171].

In a parallel development, [EMIM][TFSI] was embedded in a PVDF‐HFP matrix to fabricate a fully solid‐state ionogel with a pyramid‐shaped microstructure (Figure 5k) [173, 174, 175, 176]. Used as the gate dielectric in PEDOT:PSS and poly(3‐hexythiophene‐2,5‐diyl) (P3HT) based OECTs, this system enabled precise pressure sensing, showcasing how IL‐enhanced devices can also address mechanosensory modalities such as tactile perception. In the uncompressed state, the limited contact area restricted ion injection and capacitance. Under applied pressure, contact area and gate/electrolyte capacitance (C_G_) increased beyond the electrolyte/channel capacitance (C_CH_), facilitating enhanced doping/dedoping of the channel by EMIM^+^ cations (Figure 5l) [177, 178, 179, 180]. Consequently, the source–drain current (I_DS_) varied sensitively with pressure (Figure 5m). The system also allowed for gate bias‐dependent sensitivity tuning and demonstrated accurate detection of subtle physiological signals such as wrist pulses, confirming its potential as a high‐performance wearable biosensor (Figure 5n) [173].

Beyond signal detection, ILs are also being investigated for biodegradable and biocompatible bioelectronics. While many conventional ILs used in bioelectronics have shown cytotoxicity or raise biosafety concerns upon direct tissue contact, among various IL candidates, choline‐based ILs (e.g., [Ch][MA] (choline cations and malate anions)) offer distinct advantages due to their derivation from naturally occurring molecules [181, 182, 183]. These ILs exhibit low cytotoxicity, compatibility with metabolic degradation pathways, and tunable interactions with biological tissues [184, 185]. When combined with polysaccharide matrices such as levan, they produce electrolytes with high adhesiveness and softness, suitable for transient or implantable biomedical applications [186, 187, 188]. A notable example is the levan‐based solid‐state electrolyte (LSE), which simultaneously served as both the dielectric and substrate in an organic transistor [15]. The device employed poly[3‐(5‐carboxypentyl)thiophene‐2,5‐diyl] (P3CPT) as the semiconducting layer and gold as the electrode (Figure 5o) [189]. The ∼100 µm thick levan/[Ch][MA] film adhered directly to biological tissues without additional conductive gels. The device operated in two regimes depending on the applied voltage, with electrostatic gating occurring below 0.5 V and ion injection‐driven OECT‐like behavior emerging above 0.5 V (Figure 5p) [14, 190, 191]. When applied to a live rat heart, the device successfully recorded real‐time ECG signals with peak currents around 15 µA. The signal gradually diminished over time due to biodegradation of the electrolyte in response to bodily fluids, confirming the functionality of the intended degradation mechanism [192, 193, 194]. Unlike conventional Ag/AgCl electrodes that require conductive gels, the soft and adhesive LSE film enables direct contact with tissue, serving as both dielectric and substrate [195]. This illustrates the promise of IL‐based bioelectronics in transient or single‐use in vivo applications, wherein the device operates reliably and then safely degrades without leaving toxic residues [196].

Recent studies have demonstrated that a deep understanding of the physicochemical properties of ILs enables systematic optimization of device performance [130, 197, 198]. Changes in ion transport and interfacial behavior during operation directly affect device performance, and these effects become particularly critical in applications requiring stable detection and amplification of biological signals [157]. Accordingly, ILs have been introduced into both the channel and electrolyte of OECTs to improve device performance (Figure 5q). At the channel level, infusion of a choline‐based IL into PEDOT:PSS induces ion–polymer interactions, resulting in partial dedoping and redistribution of charge carriers. These modifications shift the transconductance (*g_m_*) peak toward lower gate voltages (*V_GS_*) and enable efficient device operation within voltage ranges suitable for low‐amplitude biological signals (Figure 5r). At the electrolyte level, the [Ch][Ace] gel (choline acetate ionic liquid gel, CAGel) leverages the hygroscopic and ionic conductive nature of the choline‐based IL to achieve improved stability compared to conventional hydrogel electrolytes. The combination of CAGel, which suppresses water loss while maintaining ionic conductivity, and an ion‐infused channel enables the OECT to sustain stable signal amplification for up to 3 months (Figure 5s) [157]. These results suggest that ILs are key materials capable of simultaneously controlling device performance and stability, holding significant implications as a material design strategy for bioelectronic sensors requiring long‐term operation.

Ionic liquids have transcended their initial role as ionic conductors and have evolved into multifunctional components that serve as structural reinforcers, ion transport regulators, dynamic dopants, and biocompatible interface materials. Their tunable chemical structures, interface adaptive properties, and electrochemical robustness position ILs as key enablers in the advancement of next‐generation biosignal interfacing electronics, offering new opportunities in wearable, implantable, and environmentally sustainable bioelectronic systems.

Despite their strong potential, several challenges remain for integrating IL‐based components into real‐world bioelectronic devices. At the device level, long‐term operational stability under physiological conditions is a primary concern. In gel‐based systems, evaporation, leakage, and gradual chemical degradation during prolonged operation can lead to performance deterioration. In addition, the inherently hygroscopic nature of many ILs may induce drift in ionic conductivity and interfacial properties unless appropriate encapsulation or stabilization strategies are employed [199, 200]. These material instabilities directly translate into device‐level electrical performance drift, including changes in transconductance, threshold voltage shifts, and slower responses, making commercial‐grade reproducibility cannot be achieved. Addressing these issues is therefore essential for enabling reliable and stable operation of IL‐based bioelectronic sensors and transistors beyond laboratory‐scale demonstrations.

#### 4.1.2 Motor Output Interface: Actuators and Soft Robotics

Bioelectronics is evolving beyond simple detection or transduction of biological signals toward systems capable of actively responding to biological inputs and physically interacting with living environments [201, 202, 203, 204, 205]. Within this trend, actuator‐based soft robotics has emerged as a key technology for next‐generation biointerfaces that directly engage with biological systems [206, 207, 208, 209]. These actuator‐integrated platforms offer functional interfaces capable of generating mechanical outputs in response to electrical or chemical stimuli in a flexible and adaptive manner [210]. Such systems can replicate muscle‐like motion or serve as feedback‐controlled therapeutic platforms that modulate mechanical stimulation in response to biological cues [211, 212, 213]. However, the practical realization of these interfaces requires materials that can deliver high ionic transport efficiency while operating under low voltage conditions [214, 215].

ILs have been widely adopted as electrolytes for soft actuators due to their advantageous properties, including high thermal stability, low volatility, wide electrochemical windows, and the ability to adjust cation and anion species independently [216, 217]. In particular, [EMIM][BF_4_] exhibits both ionic size asymmetry and high mobility, offering favorable electrochemical characteristics for the formation of electrical double layers (EDLs). The reversible accumulation and movement of ions drive the contraction–expansion deformation of actuators, and these ion dynamics interact closely with electrode design and interfacial structure [218, 219, 220, 221]. Accordingly, recent studies have proposed materials and design strategies based on ILs, with a focus on optimizing structures of the electrode and modulating interfacial ion interactions to overcome fundamental limitations in ion transport.

**FIGURE 6:**
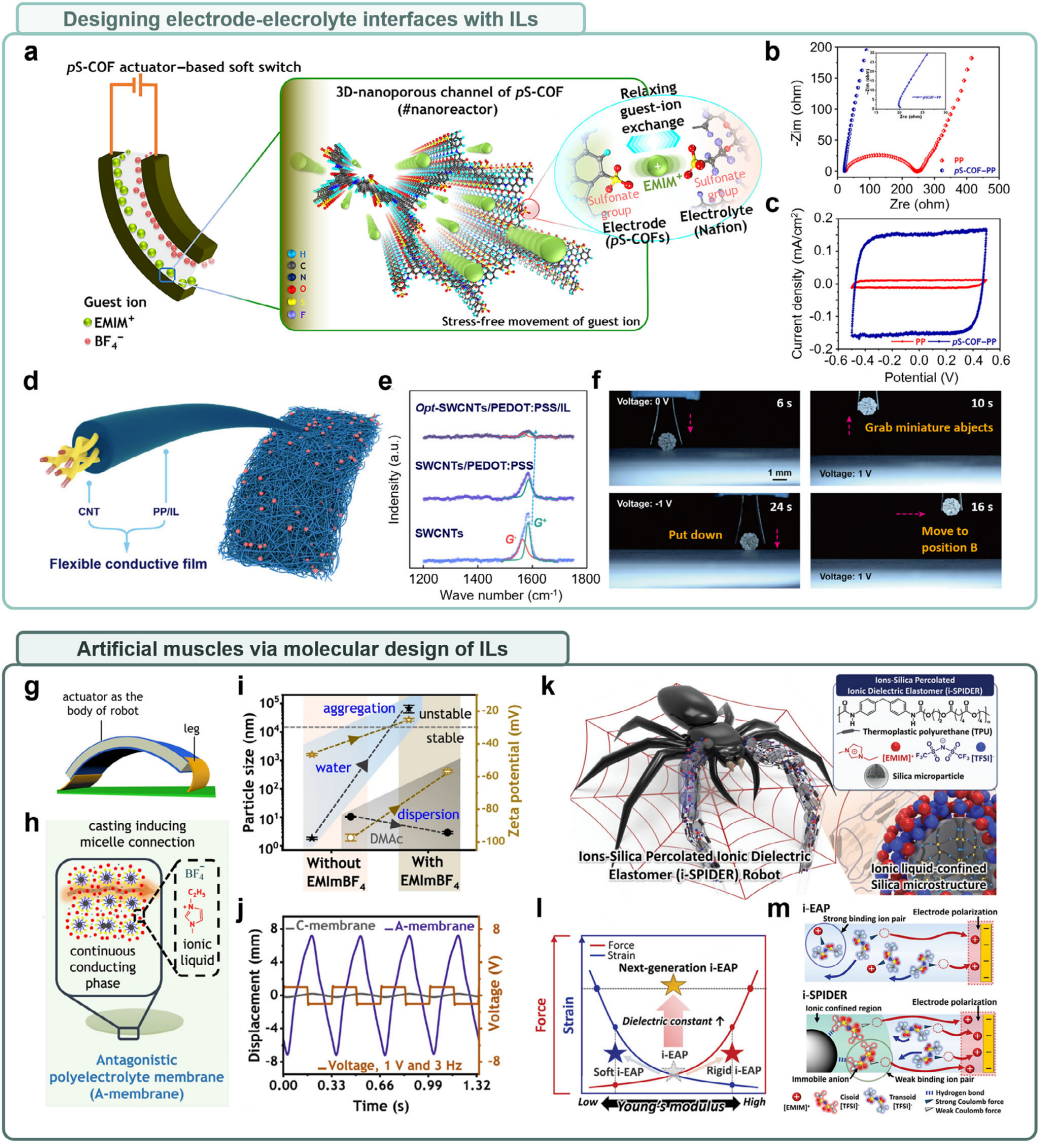
Examples of ion transfer for stimulation interface: actuators and soft robotics based on ionic liquids. (a) Schematic of the internal structure and working principle of a soft electrochemical switch utilizing polysulfonated covalent organic frameworks (pS‐COFs) as a common electrode–electrolyte host. common host platform toward relaxed mechanical bending under ultralow electrical field. The 3D nanoporous channels of pS‐COFs promote efficient ionic conduction under ultralow electric fields, while sulfonate moieties enable relaxing exchange of guest ions at the interface of both electrode and electrolyte, facilitating mechanical relaxation during actuation. b,c) Electrochemical and actuation performances of common host‐triggered pS‐COFs. (b) Electrochemical impedance spectroscopy (EIS) plots of PEDOT‐PSS only (PP) and pS‐COFs active material (pS‐COF–PP) based electrochemical soft actuators, demonstrating improved ionic transport properties enabled by the pS‐COF network. Inset: magnified EIS plot of pS‐COF–PP. (c) Cyclic voltammetry (CV) curves of both actuator types, showing a 12‐fold enhancement in areal ionic capacitance for the pS‐COF‐PP actuator. This indicates significantly improved electrochemical energy storage and actuation performance, enabled by the ionic liquid‐compatible, unified ion transport framework. Reproduced with permission [222]. Copyright 2023, The American Association for the Advancement of Science. (d) Schematic of the multilayer fiber‐type ternary electrode system. (e) Raman spectra of the sequential surface modifications of the ternary electrode. The rightward shifts in peak positions after PEDOT:PSS functionalization and IL coating on SWCNTs indicate enhanced charge transfer interactions, confirming improved electrochemical responsiveness. (f) Demonstration of a two‐claw gripper, capable of delicately grasping the microlens, indicating its potential in precision micro‐manipulation and soft robotic applications. Reproduced with permission [229]. Copyright 2025, John Wiley and Sons. (g) Application of ionic actuators for use in an inchworm‐inspired soft robot. (h) Fabrication of Nafion micelles and ionic liquid‐based polyelectrolyte membrane (A‐membrane). Nafion forms micelles in a mixed solvent system, and [EMIM][BF_4_] ionic liquid promotes micelle aggregation by interacting with the hydrophilic side chain, enabling the formation of a stable actuator membrane. (i) Particle size and zeta potential data confirm IL‐induced micelle aggregation. (j) Demonstration of typical actuator bending under 1 V and 3 Hz stimulation. Reproduced under terms of the CC‐BY license [237]. Copyright 2024, The Authors, published by Springer Nature. (k) Schematics of the arachnid‐inspired i‐SPIDER robot for soft robotic applications. The top inset shows the chemical structures and components layout of i‐SPIDER films, including the polymer matrix, IL, and silica microparticles. The bottom inset depicts the IL‐confined silica microstructure, a key architecture enabling enhanced electroactuation. (l) Conceptual comparison of the actuation performances (strain and force) among the soft, rigid, and next‐generation ionic electroactive polymer (i‐EAP), plotted against Young's modulus and dielectric constant. The i‐SPIDER system achieves an optimized balance for soft, high‐output actuation. (m) Schematic representation of the ion behaviors in i‐EAP and i‐SPIDER under increasing electrode polarization. In i‐SPIDER, reduced ionic interaction promotes enhanced ion accumulation near electrodes, leading to improved actuation displacement and force generation. Reproduced under terms of the CC‐BY license [238]. Copyright 2023, The Authors, published by John Wiley and Sons.

To address the ion transport bottleneck at the electrode–electrolyte interface, a nanoreactor‐based electrode (polysulfonated covalent organic framework, pS‐COF actuator) was developed to provide stress‐free ion migration pathways (Figure 6a) [222, 223, 224, 225]. This architecture effectively solved the intrinsic limitations of IL‐based systems, such as ion trapping and diffusion delays, through the incorporation of sulfonate moieties [226, 227]. The porous electrode structure functionalized with sulfonate groups electrostatically captures cations (EMIM^+^), forming a relaxing conduction path that facilitates high charge transfer efficiency and stable ion flow. Electrochemical impedance spectroscopy (EIS) further confirms a substantial reduction in charge transfer resistance (R_ct_), as evidenced by the Nyquist plot (Figure 6b), supporting enhanced electrode–electrolyte interactions and improved ion transport dynamics [228]. The resulting pS‐COF‐PP soft actuator demonstrated a 12‐fold increase in charge storage capacity compared to conventional IL‐based actuators (Figure 6c), along with markedly improved multifunctional mechanical performance, including faster response time, greater blocking force, and higher bending stress [222]. This work clearly demonstrates the impact of interfacial ion transport regulation on overall device performance.

Focusing on the enhancement of ion insertion and extraction efficiency within the electrode, a high‐performance soft actuator was engineered using a core–shell ternary structure composed of carbon nanotubes (CNTs), PEDOT:PSS, and the ionic liquid [EMIM][BF_4_] (Figure 6d) [229, 230, 231]. This architecture was designed to maximize electrochemical sensitivity while ensuring high electrical conductivity and mechanical durability [232, 233]. The surface coating of PEDOT:PSS and IL on the electrode significantly contributed to electrochemical responsiveness, as evidenced by a frequency shift of the Raman spectral peak toward the higher wavenumber region, indicating improved charge transport within the CNT electrode (Figure 6e). Upon voltage application, asymmetric expansion is induced due to the size difference between EMIM^+^ and BF_4_^−^ ion pairs, enabling precise bending motions [234]. This mechanism was further validated through the demonstration of a functional micro gripper, highlighting its potential applicability in real‐world tasks (Figure 6f) [229]. Such a compliant actuation strategy offers strong suitability for bioinspired robotic systems or micromechanical devices, particularly in tasks requiring delicate manipulation, such as grasping fragile objects [235, 236].

In summary, the high performance of IL‐based soft actuators requires a sophisticated integration of electrode–electrolyte interface engineering and electrode structural design, which effectively controls material‐level ion dynamics, significantly expanding their application potential in biomimetic artificial muscles and precision robotic systems (Figure 6g,k) [237, 238, 239, 240, 241, 242, 243]. Achieving this level of performance further necessitates the design of electrolytes that can simultaneously ensure selective ion transport and structural stability [215, 219, 220, 244]. However, ionic conductivity typically originates from hydrophilic side chains, whereas mechanical robustness is largely derived from hydrophobic domains, making it structurally challenging to achieve both properties simultaneously in conventional electrolyte systems [245, 246]. Consequently, novel molecular design strategies are needed to simultaneously realize efficient ion transport and mechanical stability.

Against this background, a recent strategy has been proposed to fabricate micelle‐based polyelectrolyte membranes by exploiting the electrostatic equilibrium modulation capability of ILs [247, 248, 249, 250]. This approach not only ensures the continuity of ion transport pathways but also significantly enhances multiple performance metrics of the actuator (Figure 6h). The strategy is based on the control of the microstructure in solution through the IL‐mediated electrostatic equilibrium [251]. Specifically, upon the introduction of ILs, the dielectric constant of the system is reduced by the water‐soluble salts, thereby decreasing the coulombic repulsion between similarly charged ions, leading to aggregation among micelles (Figure 6i) [244, 248, 252]. This process facilitates the formation of a continuous membrane that achieves both high mechanical stability and conductivity while maintaining a hydrophobic backbone. The actuator based on this antagonistic polyelectrolyte membrane (A‐membrane) exhibited more than a 35‐fold enhancement in bending response under low driving voltage (1 V) and high frequency operation (3 Hz), with performance sustained across a broad frequency range from 0.01 to 20 Hz (Figure 6j) [237]. This behavior results from rapid ion transport, which is facilitated by the molecular‐level ionic conduction network formed within the membrane under an applied electric field [222, 253]. As a result, the system demonstrated improvements across various performance parameters, including shortened response time, suppression of relaxation effects, low operational voltage, wide bandwidth, long‐term stability, and high blocking force [254, 255, 256]. Furthermore, this system is applied to a soft robot with inchworm motion to demonstrate its practical applicability [237].

Ionic electroactive polymers (i‐EAPs) have shown promising potential as soft actuators for human–machine interface applications [257, 258, 259]. However, their performance has remained limited due to an intrinsic trade‐off between high output force and large strain (Figure 6l) [260, 261, 262]. In particular, the formation of EDLs through ion accumulation at the electrode–electrolyte interface restricts actuation force, and the balance between ion dissociation and dielectric constant has been identified as a critical parameter influencing device performance [263, 264, 265]. To overcome these limitations, an ions‐silica percolated ionic dielectric elastomer (i‐SPIDER) structure was developed by combining the IL [EMIM][TFSI] with silica nanoparticles. In this system, partial confinement of the IL onto the silica surface weakens the electrostatic binding between ion pairs, thereby promoting ion dissociation and enhancing interfacial polarization (Figure 6k) [266, 267]. Notably, TFSI^−^ anions transition from the transoid to cisoid conformer, which facilitates the formation of aligned ion pathways and maximizes ion accumulation efficiency under electric fields (Figure 6m) [266, 268, 269]. Simultaneously, this architecture improves the dielectric constant while suppressing charge leakage, enabling simultaneous enhancement of both electrical performance and mechanical stability [238]. By overcoming structural limitations inherent to conventional i‐EAPs, this strategy successfully demonstrated precise biomimetic robotic functions, including insect‐like crawling, object manipulation, and posture control, thus highlighting its potential for next‐generation, low‐power soft robotic systems [270, 271, 272].

Soft robotics based on organic electronic devices and ionic liquid‐driven actuators represents a key technology capable of shifting the paradigm of future biointerfaces from passive sensing to active interaction. Despite this potential, the integration of ionic liquid‐based actuation with organic electronic systems in biointerface research remains at an early stage. Further fundamental studies are required to clarify the diverse transport mechanisms associated with various types of ionic liquids and to evaluate their stability under physiological conditions. Moreover, the design of fully integrated soft systems that combine sensing, stimulation, and feedback functionalities remains both a significant challenge and a promising direction for future research.

### 4.2 Bio‐Integration Interface: Wearable and Implantable Devices

Wearable and implantable bioelectronic devices are enabling a new paradigm of personalized healthcare through their exceptional mechanical properties, compatibility with biofluids such as sweat, real‐time biosignal detection, signal visualization, and remote monitoring capabilities [273, 274]. In the advancement of these bioelectronic devices, ILs have expanded their role beyond flexible electrolytes to include use as functional structural materials. Leveraging their intrinsic stretchability, chemical stability, and tunable electrochemical properties, IL‐based bioelectronic interfaces are increasingly being integrated into wearable and implantable platforms. These systems are now evolving into multifunctional sensors capable of operating in biofluid‐rich and chemically dynamic environments, thereby supporting complex biosignal acquisition and long‐term physiological monitoring.

**FIGURE 7:**
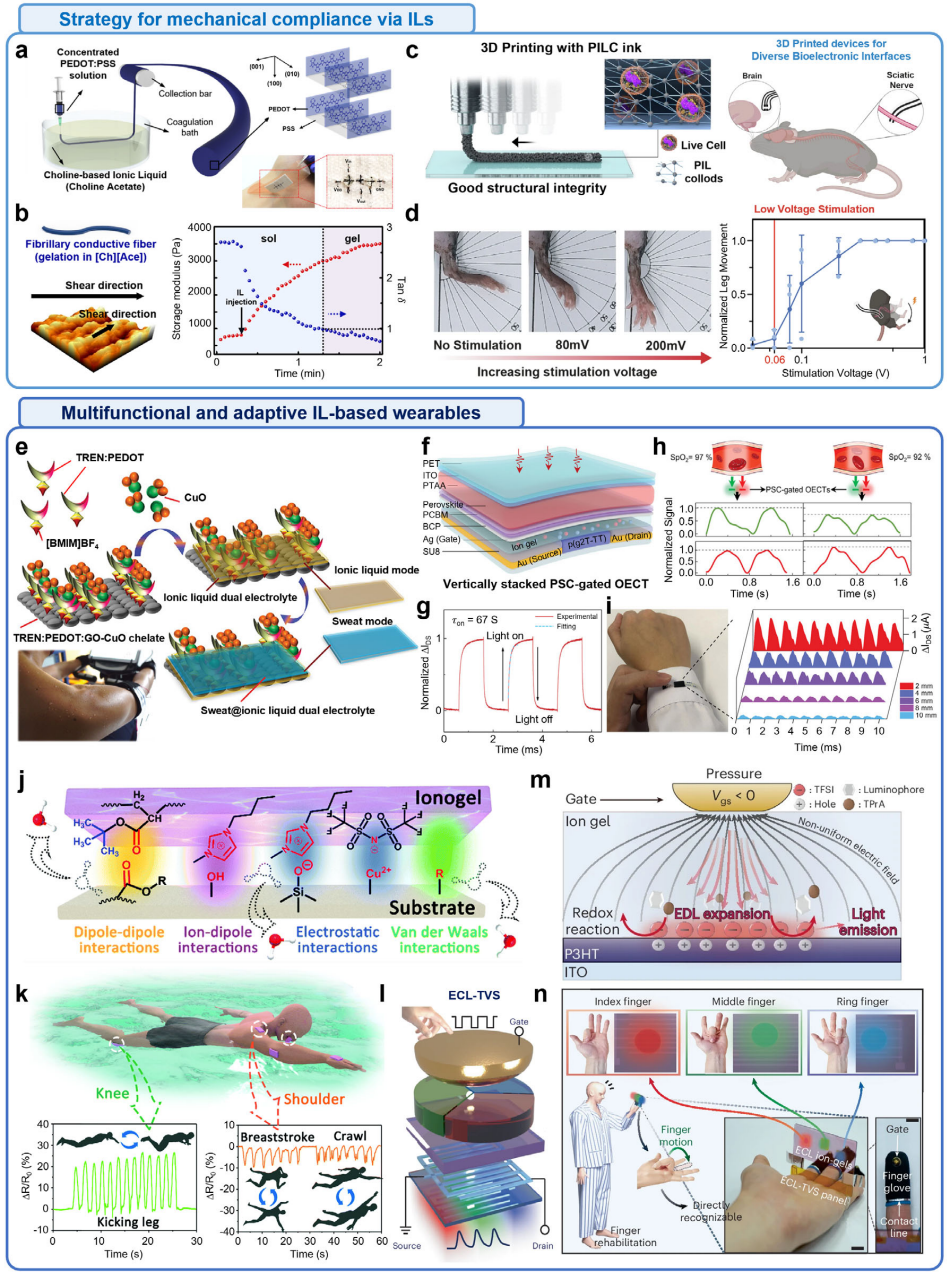
Bio‐integration interface: wearable and implantable devices based on ionic liquids. (a) Schematic illustration of fibrillary gelation initiated by injecting concentrated PEDOT:PSS solution into an ionic liquid (left), and resulting molecular alignment of the fabricated fibers (right). (b) AFM image showing molecular alignment induced by shear force during fiber fabrication (left). Storage modulus increase and tan δ decrease, confirming mechanical stability during sol‐to‐gel transition (right). Reproduced under terms of the CC‐BY license [278]. Copyright 2022, The Authors, published by Springer Nature. (c) Fabrication of biocompatible 3D printing inks using PEDOT:PSS‐ionic liquid colloids (PILC), demonstrating structural integrity and biocompatibility with live cells (left). Illustration of a fabricated device applied for sciatic nerve stimulation in mice (right). (d) Performance evaluation of the PILC‐based sciatic nerve stimulation device (left). Quantitative data of leg movement displacement according to applied stimulation voltage (right). Reproduced under terms of the CC‐BY license [283]. Copyright 2024, The Authors, published by Springer Nature. (e) [BMIM][BF_4_]^−^ based dual electrolyte supercapacitor integrated with sweat, demonstrating self‐charging functionality when worn on the wrist. Reproduced under terms of the CC‐BY license [286]. Copyright 2022, The Authors, published by John Wiley and Sons. (f) Device structure of vertically stacked PSC‐gated organic electrochemical transistor (OECT). (g) Ultrafast response (67 µs) shown by current–time profiles under pulsed illumination (on/off). (h) Photoplethysmogram (PPG) signals obtained using red and green light wavelengths for accurate monitoring of blood oxygen saturation (SpO_2_). (i) Demonstration of remote PPG signal detection and rapid response measured at distances up to 10 mm from the wrist. Reproduced with permission [289]. Copyright 2022, John Wiley and Sons. (j) Real‐time, precise monitoring of underwater respiration and body movements through sensors attached to the human cheek, knee, and shoulder. (k) Schematic illustration showing adhesion mechanisms between various substrates and ionic liquid‐based ionogels, emphasizing multiple non‐covalent interactions ensuring robust adhesion even underwater. Reproduced with permission [158]. Copyright 2014, Royal Society of Chemistry. (l) Detailed structure of electrochemiluminescent tactile visual synapse (ECL‐TVS) device, illustrating sensor operation through pressure (gate) input and its multilayered configuration (ion‐gel, P3HT channel, electrodes). (m) Demonstration of electrochemiluminescence (ECL) signals triggered by tactile inputs from fingers, showing potential application in finger rehabilitation and real‐time monitoring. (n) Mechanism illustrating asymmetric electric field formation and electrical double layer (EDL) expansion induced by applied pressure (V_G_ < 0). Reproduced with permission [299]. Copyright 2025, Springer Nature.

To ensure seamless integration with soft biological systems, it is essential to engineer bioelectronic materials with tunable mechanical properties and structural adaptability [275, 276, 277]. In this regard, ILs can function as morphological and mechanical modulators within conducting polymer matrices. For example, high‐performance fiber‐based OECTs were fabricated using a choline acetate‐based IL, which induces fibrillary gelation of PEDOT:PSS (Figure 7a) [278]. The IL facilitates this process by employing its high ionic strength to extract water from the polymer matrix, while simultaneously dissolving PSS chains and effectively weakening the electrostatic interactions between PEDOT and PSS. This, in turn, induces PEDOT chain alignment and phase separation (Figure 7b) [279, 280, 281]. This gelation process significantly enhances mechanical stability and electrical conductivity [282]. Consequently, the fiber‐based OECT exhibited a high elongation of 29.5%, a value comparable to that of typical textile fibers, and maintained excellent durability after 10 000 repeated bending cycles. Extending this IL‐based gelation technique, a biocompatible PEDOT:PSS‐IL colloid (PILC) based on 1‐ethyl‐3‐methylimidazolium tetracyanoborate ([EMIM][TCB]) was developed as a highly conductive (∼286 S/cm) 3D printing ink, surpassing conventional PEDOT:PSS conductivity (Figure 7c) [283, 284, 285]. This ink enabled instant biocompatibility and high‐resolution 3D printing without post‐treatment and clearly demonstrated increased leg movements in sciatic nerve stimulation experiments conducted on mice at stimulation voltages of 80 mV and 200 mV (Figure 7d). These results suggest the potential of ILs in controlling structural, electrical, and mechanical properties of fiber architectures simultaneously, allowing direct integration into wearable textile electronic devices as logical and information processing components. Thus, the fibers demonstrated practical applicability when integrated onto wearable substrates such as bandages or clothing, or as implantable bioelectronics interfaces.

To explore practical wearable applications, an IL‐based supercapacitor electrode was developed using [BMIM][BF_4_], which forms a dual electrolyte system when combined with sweat (Figure 7e) [286]. In this configuration, the IL interacts with sweat ions to establish a complementary electrolyte environment. Specifically, the IL facilitates EDL formation and excellent ionic diffusion characteristics, enabling simultaneous and synergistic ionic and charge transport pathways at the electrode interface under sweat exposure [287]. This dual‐electrolyte mechanism significantly enhances electrochemical performance (areal capacitance of 3600 mF/cm^2^, energy density of 450 mWh/cm^2^) compared to single electrolyte systems. When integrated with tris(2‐aminomethyl)amine:PEDOT/graphene oxide/copper oxide (TREN:PEDOT:GO/CuO) chelate composites, the resulting flexible supercapacitor demonstrates high energy density and long cycle life even in the absence of enzymatic components [288]. Real‐world testing further confirms that the wearable device, when attached to the wrist, can actively utilize sweat to store electrical energy, verifying the applicability of ILs as biointerfaces for self‐charging energy storage via biofluid harvesting.

Beyond direct skin contact, next‐generation wearable biointerfaces aim to enable comfortable and noninvasive monitoring by integrating optoelectronic sensing with high sensitivity. To address this need, a vertically stacked high‐performance flexible photodetector integrating perovskite solar cells (PSC) and OECT was also developed (Figure 7f) [289]. In this study, an [EMIM][TFSI]‐based solid electrolyte (ion gel) effectively transferred the photovoltage generated by PSC illumination to the OECT channel, achieving precise and rapid current modulation via high ionic conductivity. The resulting structure exhibited high sensitivity and ultrafast response (67 µs) (Figure 7g), enabling accurate real‐time monitoring of blood oxygen saturation (SpO_2_) (Figure 7h) and remote photoplethysmography (PPG) measurement at distances up to 10 mm from the body (Figure 7i) [290, 291]. This research confirms ILs as critical mediators for optical and bio signal sensing in wearable healthcare monitoring applications.

Meanwhile, to address ion leakage and swelling issues typically encountered with conventional ionogels in underwater environments, a fully hydrophobic ionogel based on [BMIM][TFSI] was designed [158, 292, 293, 294]. ILs facilitated robust adhesion by forming various non‐covalent interactions such as dipole–dipole, ion–dipole, electrostatic, and van der Waals forces between diverse substrates (glass, plastic, silicone, textiles, metals, rubber, and skin) and the ionogel (Figure 7j) [158, 160, 295, 296, 297, 298]. In particular, the strong hydrophobicity and multiple intermolecular interactions provided effective blocking of moisture and ion penetration, suppressing swelling and ion leakage, and destroying hydration layers commonly formed in underwater conditions, thus maximizing substrate–ionogel adhesion. The ionogel demonstrated exceptional durability over five months, with a remarkable elongation of 1145% and Young's modulus of 115.6 kPa, maintaining superior mechanical and electrical stability. Leveraging these structural and chemical designs, the sensors attached to various human body parts, including cheeks, knees, and shoulders, accurately monitored underwater respiration, limb movements, and swimming stroke types in real‐time experiments (Figure 7k). This IL‐based hydrophobic ionogel demonstrated stable sensing and adhesion performance on diverse substrates and under challenging conditions, offering a practical solution for underwater physiological monitoring and paving the way for future applications in wearable safety and rescue technologies.

Advanced IL‐based ionogels have further been demonstrated in electrochemiluminescent tactile visual synapses (ECL‐TVS), enabling simultaneous tactile stimulation sensing and electrochemiluminescence (ECL) visualization (Figure 7l) [299, 300, 301]. Ion gels designed with 1‐butyl‐1‐methylpyrrolidinium bis(trifluoromethylsulfonyl)imide ([PYR_14_] [TFSI]) ILs exhibited immediate responses in both color‐based light emission (red, green, blue) and electrical signals according to tactile input pressures (Figure 7n). Specifically, as shown in the figure, when pressure (negative gate voltage) was applied, an asymmetric electric field formed between gate and channel, inducing penetration of TFSI^−^ anions into the active layer (P3HT channel), expanding the EDL. This EDL expansion subsequently triggered redox reactions between luminophores and co‐reactants tripropylamine (TPrA), resulting in immediate visible light emission (Figure 7m). Benefiting from excellent ionic conductivity and high chemical stability, these ionogels operate under ultralow power (<34 µW), demonstrating a novel class of wearable synaptic devices capable of simultaneous visual and electrical tactile feedback as advanced biointerfaces.

Collectively, these advancements underscore the multifaceted role of ILs in advancing biointegrated electronics. By enabling precise control over mechanical compliance, interfacial adhesion, and electrochemical transduction, ILs serve as critical design elements for achieving seamless integration with biological systems. Their inherent tunability facilitates adaptive matching between synthetic devices and soft, dynamic tissues, while supporting reliable signal transmission, energy storage, and long‐term operational stability. As such, ILs not only enhance the functional performance of wearable and implantable platforms but also establish a versatile material framework for next‐generation bioelectronic interfaces that operate across complex, physiologically relevant environments.

ILs are primarily integrated and utilized in solid‐state form rather than as a neat liquid state for practical electronic applications. This is because the intrinsically liquid nature of ILs leads to limitations in terms of leakage, morphological instability, processability, and device integration, and long‐term stability is difficult to achieve through simple encapsulation alone. A key strategy to overcome these limitations is gelation. Ionogels, which immobilize ILs within a polymeric or inorganic matrix, are an intermediate state of matter that combines the flexibility and ionic mobility of liquids with the shape stability and mechanical strength of solids. As a result, they are particularly well‐suited for electronic device integration. From an electronic application perspective, the performance of IL‐based gels strongly depends on the choice of matrix, which is not arbitrary but is determined by a combination of the physicochemical properties of the IL's polarity, ion size, hydrophilicity/hydrophobicity, and electrochemical stability, and device requirements. Accordingly, numerous studies have reported the integration of ILs with a wide range of polymer matrices, such as fluoropolymers, polyethers, and acrylic polymers [15, 302, 303, 304, 305]. Each of these systems realizes a different balance among miscibility, ionic transport behavior, and electrochemical stability. These ionogels have been widely utilized as solid electrolytes or gate dielectrics in flexible electronic devices, electric double‐layer transistors, and energy storage systems. In addition, the gel form enables further functional extensions, including skin‐like conformability and tactile sensing capabilities through microstructural design.

Consequently, ILs alone exhibit inherent limitations in practical applications, and matrices also cannot provide functionality beyond structural support. In contrast, gel‐based systems that integrate both components show synergistically enhanced chemical, mechanical, and electrical properties. This synergy enables further expansion toward advanced functional materials such as organic mixed ionic electronic conductors (OMIEC) that are capable of ion–electron signal conversion [154, 306]. Therefore, ILs and gel‐based structures should be regarded as a unified material platform and studied together in order to fully realize their potential in electronic and bioelectronic applications.

### 4.3 Neuromorphic Interface Enabled by Ionic Liquids

All living organisms operate on complex neural network systems that respond to external stimuli, process information, and make decisions. At the core of these systems lies the brain, which governs higher‐order biological functions such as perception, learning, memory, and motor control. The entire body is interconnected through the central and peripheral nervous systems, with this connectivity mediated by a range of biological components and dynamics, including neurons, synapses, neurotransmitters, and ion transport. In particular, the electrochemical signal transduction mechanism is based on the membrane potential established across the cell membrane, and information is transmitted and modulated through the generation of action potentials and the subsequent chemical transmission processes. This sophisticated and adaptive mode of biological information processing enables fast and energy‐efficient signaling through multiple synaptic connections and spatiotemporally coordinated ion flows between neurons. Such capabilities of biological systems are increasingly being recognized as a model for overcoming the limitations of conventional digital computing architectures, especially in addressing challenges related to high computational power consumption and inefficiencies in parallel processing [307].

Against this background, neuromorphic electronics aim to emulate the operating principles of biological neural circuits to develop systems capable of low‐power, adaptive, and parallel information processing, similar to the human brain. To achieve this, extensive research has focused on ion‐based electronic devices, electrochemical transistors, and synaptic devices that reproduce the structure and function of biological synapses [308, 309]. A critical requirement for implementing neuromorphic systems is the use of materials that can mimic the ion‐based signal transduction mechanisms inherent to biological nervous systems [310, 311]. In this context, ILs have emerged as promising materials due to their ability to effectively simulate the ion exchange processes observed in biological synapses. Through ion accumulation, charge transport, and ion‐electron coupling, ILs can reproduce essential synaptic behaviors such as long‐term potentiation (LTP), long‐term depression (LTD), and spike timing‐dependent plasticity (STDP) [308]. Furthermore, the formation of high ionic concentration gradients at interfaces and the alignment of ionic pathways within dielectric matrices enable the design of devices that feature low voltage operation, high sensitivity, and excellent mechanical flexibility.

#### 4.3.1 Closed‐Loop Sensorimotor Integration

**FIGURE 8:**
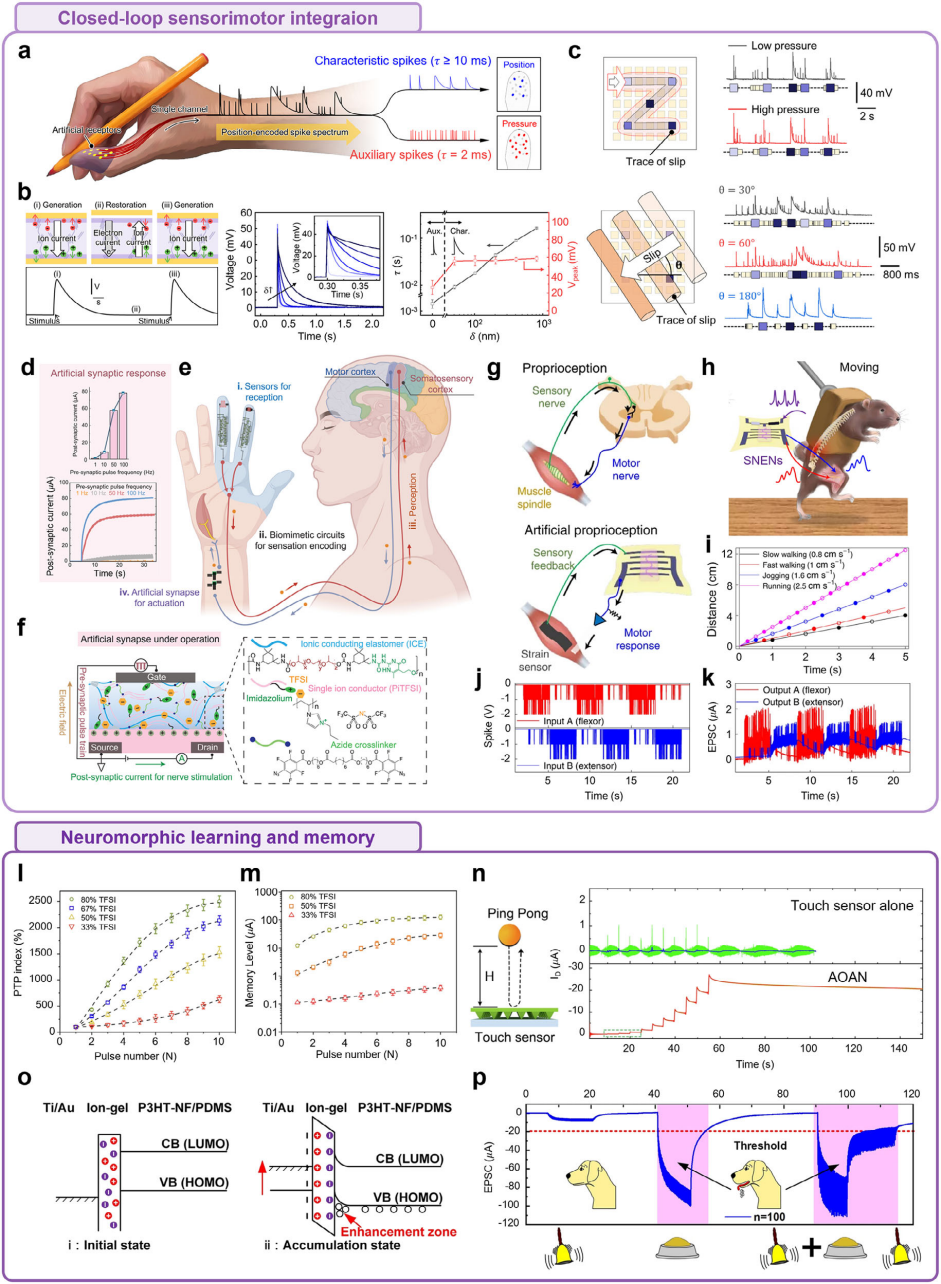
Neuromorphic interface enabled by ILs for integrated systems. (a) Scheme of the artificial receptors generating position‐ and pressure‐encoding sensory spikes, including characteristic position‐encoding spikes and auxiliary pressure‐encoding spikes. (b) Ionic and electronic transport mechanisms involved in spike generation and recovery (left). Representative electrical responses and spike profiles from artificial receptors under various δ values, with enlarged views shown in the inset (center). Extracted time constant (τ) and peak voltage (V_peak_) of the spikes as a function of δ, fitted with exponential decay [V = V_peak_∙exp(−t/τ)] (right). (c) Dynamic tactile sensing performance. Schematic of slip and draw stimulation (top‐left). Spatial distribution of contact area under low (black) and high (red) pressure, with corresponding position‐encoded spike spectrum (PESS) signals (top‐right). Slip stimuli at various angles (θ = 30°, 60°, 180°) using a cylindrical object and the corresponding spike responses (bottom). Reproduced with permission [315]. Copyright 2022, The American Association for the Advancement of Science. (d) Frequency‐dependent response of an artificial synapse, showing increased postsynaptic current with higher presynaptic pulse frequency. (e) Biointegrated e‐skin system forming an artificial sensorimotor loop: sensory receptors encode external stimuli into spike trains via low voltage circuits, and artificial synapses trigger muscle‐like actuation. (f) Structure and components of an artificial synaptic transistor mimicking biological synapse components. Reproduced with permission [325]. Copyright 2023, The American Association for the Advancement of Science. (g) Schematic of a stretchable neuromorphic efferent nerve (SNEN) using organic synaptic transistors to bypass damaged nerves and relay signals to muscles for locomotion. (h) Mouse demonstrating recovered voluntary locomotion through SNEN‐mediated, coordinated stimulation of muscles. (i) locomotion distances achieved at different movement speeds (0.8–2.5 cm/s). j,k) Presynaptic voltage spike (j) and corresponding EPSCs (k) from synaptic transistors. Reproduced with permission [331]. Copyright 2022, Springer Nature. (l) Synaptic behavior emulated by an artificial organic afferent nerve (AOAN) device: Post‐tetanic potentiation (PTP) index plotted against the number of input pulses for different ion blend ratios. (m) Memory retention level as a function of pulsed pressure number (N), indicating the transition from short‐term plasticity (STP) to long‐term potentiation (LTP). (n) Enhancement of tactile sensitivity to subtle stimuli through repeated training. Reproduced with permission [347]. Copyright 2024, Springer Nature. (o) Working mechanism of the tribotronic artificial synapse (TAS) illustrated via energy band diagrams under mechanical stimulation. (p) Simulation of associative learning analogous to Pavlov's experiment, showing EPSC responses of TAS device modulated by varying training time (*n* = 100). Reproduced under terms of the CC‐BY license [351]. Copyright 2022, The Authors, published by Springer Nature.

In neuromorphic systems, the ability to accurately detect stimuli and decompose them with high spatiotemporal resolution is essential for high‐fidelity sensory information processing. A recently proposed position encoded spike spectrum (PESS) based artificial dynamic sensory system mimics the event‐driven, parallel neural signal processing principles of the human somatosensory system, enabling spike‐based stimulus recognition without the need for traditional transducers (Figure 8a) [307]. Similar to human fast‐adapting type I (FA‐I) afferents, the system utilizes the relative timing of initial spikes to rapidly identify contact position, direction, and object shape, offering more decisive information than spike frequency alone [312, 313, 314]. The platform utilizes potential spikes generated in mixed ion electron conductor (MIEC) based artificial receptors, and by modulating the concentration of ionic liquids, the spike relaxation time (τ) can be tuned over a broad range from 2 to 160 ms, allowing for precise temporal encoding within the PESS framework (Figure 8b) [282, 315]. Notably, short τ spikes (∼2 ms) encode contact area, while longer τ spikes (≥ 10 ms) correspond to stimulus position. Furthermore, the spike sequence and relative timing information acquired during 2D drawing tasks enable the recognition and the discrimination of contact and slip angles, demonstrating the system's potential for precision position‐based tactile perception and its applicability in robotic sensory systems (Figure 8c) [315]. Artificial neural devices can generate distinct electrical spike patterns in response to variations in stimulus location, intensity, and frequency, thereby offering high spatiotemporal resolution for the perception and classification of complex dynamic tactile inputs [316, 317]. This enables localized learning based response control within the system. The simple sensing platform holds great promise for the development of high‐resolution, omnidirectional artificial skin that can be applied to service robotics and the understanding and restoration of human skin sensory functions.

Beyond simple sensory perception, the ability to convert tactile input into real‐time sensory feedback plays a pivotal role in neuromorphic electronic skin. By interpreting sensory inputs and generating corresponding motor outputs, such systems enable the realization of closed‐loop human‐machine interfaces [318, 319]. Despite extensive efforts to integrate multimodal sensory reception, neuromorphic signal processing, and adaptive actuation, the realization of sensorimotor interfaces that precisely merge with biological systems remains a significant challenge [320, 321, 322, 323, 324]. To address this limitation, one approach has been explored that mimics the biological principle of encoding stimulus intensity into spike frequency in somatosensory pathways [325]. When presynaptic pulses of varying frequencies were applied as synaptic inputs, the amplitude of the postsynaptic current varied by up to 10^7^‐fold (∼800 Hz) depending on the frequency, a property that is essential for precisely converting sensory strength into graded motor outputs (Figure 8d). Based on this operational mechanism, a frequency‐based artificial e‐skin system was proposed, in which sensory input is encoded in spike frequency to generate motor output (Figure 8e) [307, 321, 324, 326, 327]. This system extends beyond simple sensing, functioning as a neuromorphic e‐skin capable of forming a closed‐loop sensory‐motor circuit. Its core component is a solid‐state ionic dielectric, fabricated by blending a high ion conductive elastomer (ICE) with a single ion conducting polyelectrolyte (PiTFSI), which overcomes the stability limitations of conventional gel‐ or liquid‐based synaptic transistors (Figure 8f) [321, 328]. Using this synapse, a complete sensorimotor loop was successfully demonstrated, where sensory input originating in the somatosensory cortex of a rat was transmitted through the motor cortex to activate the sciatic nerve and induce actual muscle contraction [325].

The successful implementation of this unidirectional sensory‐motor loop represents an important starting point for the functional expansion of neural interfaces [329, 330]. However, to meet the demands of precision, responsiveness, and safety required in actual motor control, the integration of proprioception‐based feedback loops is essential. In the biological nervous system, proprioception provides real‐time information on muscle contraction, joint movement, and body position, enabling the suppression of excessive tension and facilitating coordinated motion. Moving beyond simple stimulus‐response behavior, a closed‐loop architecture was developed [331]. In this system, mechanical deformation caused by muscle movement is detected by an artificial proprioceptor, and the signal is subsequently provided as feedback to the synaptic input (Figure 8g). This system operates based on a stretchable proprioceptive feedback circuit composed of a synaptic transistor based on organic nanowires and a carbon nanotube strain sensor embedded within an ionic gel matrix. The implant generates electrophysiological stimulation from excitatory postsynaptic signals while simultaneously providing proprioceptive feedback, thereby functioning as an artificial efferent nerve [332]. Similar to the biological stretch reflex, precise regulation of excitatory and inhibitory responses is required to prevent muscle overstretching, which necessitates real‐time proprioceptive feedback [331]. Indeed, under feedback‐enabled conditions, motor performance remained stable, and muscle damage from repeated excessive contractions was effectively suppressed. By reproducing a range of gait speeds and patterns through proprioceptive feedback, the system demonstrates strong potential as a biomimetic rehabilitation platform (Figure 8h). The core component, the synaptic transistor, operates as follows. When a presynaptic gate voltage (V_G_) is applied, anions in the ion gel migrate and accumulate near the organic semiconductor nanowire, inducing a transient accumulation of holes from the source electrode into the nanowire [333, 334, 335]. This process generates an excitatory postsynaptic current (EPSC). Through this mechanism, the device produces stable and repeatable electrical responses, maintaining consistent electrical characteristics over time. In the stretchable neuromorphic efferent nerve (SNEN) system, biomimetic action potential (AP) signals are delivered to the synaptic transistor via an artificial proprioceptor, and the resulting EPSCs are applied to the muscle to induce actual movements. In implementation experiments, modulating the period of the input Aps allowed for controlled gait speeds, ranging from slow walking (0.8 cm/s) to fast running (2.5 cm/s) (Figure 8i). This motor control was achieved by applying signals in a cross pattern to two SNENs, each connected to either a flexor or an extensor muscle, enabling precise control of each muscle's contraction timing. Notably, in an experiment using real neural signals recorded from the primary motor cortex of an animal as the presynaptic input to SNEN, two separate SNENs (A and B), connected to a flexor and an extensor, respectively, successfully responded to varying neural firing rates (Figure 8j). Each device integrated these inputs to generate output EPSCs, thereby reproducing distinct leg swing angles with high precision (Figure 8k). These results demonstrate the feasibility of brain signal‐driven motor control and provide a critical foundation for the future development of neuromorphic rehabilitation devices [336]. Ultimately, this system functions as an artificial efferent nerve (SNEN) capable of receiving brain‐derived APs as input and precisely controlling the spatiotemporal dynamics of muscle movement [331]. As a biomimetic interface platform that can reproduce various gait speeds and patterns in response to motor cortex signals, it presents a promising therapeutic strategy for the rehabilitation of motor impairments caused by neurodegenerative diseases or spinal cord injuries.

#### 4.3.2 Intelligent Behavior through Synaptic Functionality

To realize intelligent biomimetic systems, it is essential to move beyond simple stimulus‐response mechanisms and incorporate learning and memory capabilities, namely synaptic plasticity, which enables responses to be modulated based on the history of stimuli. This function is particularly critical in motor rehabilitation contexts, where systems must adapt to repetitive stimuli and dynamically adjust their responses according to movement patterns. Such capability supports long‐term motor learning, automated motion control, and personalized response modulation. Neuromorphic devices that address these requirements effectively mimic biological learning mechanisms by electronically implementing synapse‐like features such as weight modulation, short‐ and long‐term synaptic potentiation (STP and LTP), and EPSCs at the device level [337, 338].

To effectively implement tunable synaptic behavior in neuromorphic devices, the role of ILs in modulating charge transport and doping dynamics must be carefully considered. Ionic liquids, when integrated into polymer matrices, form strong ion–dipole electrostatic interactions, enabling the formation of flexible solid electrolytes (SEs) with high ionic mobility and conductivity [339, 340, 341]. In organic electrochemical synaptic transistors (OESTs), ILs enable precise control of the doping state of organic semiconductors via volumetric electrochemical doping. The threshold voltage (V_th_) and transconductance (g_m_) are directly influenced by the hydrophilicity/hydrophobicity and blending ratio of the IL. The ionic composition of ILs plays a critical role in modulating the synapse‐like response characteristics of these devices. For instance, SEs containing a high TFSI ratio (80%) exhibit faster response and larger conductivity modulation (ΔG), whereas SEs with a lower TFSI ratio (33%) show slower recovery and longer relaxation time. These results suggest that IL composition governs ion doping/dedoping efficiency, diffusion kinetics, and accumulation mechanisms within the semiconductor [342, 343]. Consequently, the IL composition significantly influences memory retention time and synaptic weight modulation, serving as a key parameter for tuning postsynaptic current responses under varying external stimulus conditions. This behavior closely resembles biological synaptic mechanisms, in which neurotransmitters are released and reabsorbed in response to mechanoreceptor stimuli. The injection and extraction of ions in IL‐based systems effectively emulate short‐ and long‐term synaptic plasticity observed in biological systems [343, 344].

In particular, paired‐pulse facilitation (PPF), which refers to synaptic enhancement between two stimuli repeated with short intervals, and post‐tetanic potentiation (PTP), which is a gradual increase in synaptic weight induced by high‐frequency repetitive stimulation, are critical indicators for evaluating neuromorphic synaptic characteristics. The PPF index is typically calculated as the ratio of the second excitatory postsynaptic current (EPSC_2_) to the first (EPSC_1_), defined as PPF (%) = (EPSC_2_ / EPSC_1_)100. The PTP index quantifies the cumulative enhancement of synaptic efficacy over multiple spikes and is defined as PTP (%) = (EPSC_N_ / EPSC_1_)100. The PTP index can be quantitatively assessed based on the number of input spikes and the composition of the IL. Devices incorporating 80% TFSI exhibited a significantly high PTP index of up to 2500%, whereas devices with 33% TFSI showed a relatively low PTP of approximately 500%, with a noticeable decline in facilitation after saturation (Figure 8l). Such short‐term plasticity (STP) can evolve into long‐term potentiation (LTP) through repeated stimulation, which serves as a crucial mechanism for implementing memory formation and learning functionalities in neuromorphic devices [344, 345, 346]. Indeed, in the artificial organic afferent nerve (AOAN) device, increasing the number of pressure‐induced spikes led to a gradual rise in memory levels observed at 300 s, reaching a memory retention characteristic as high as 126 µA with 80% TFSI composition (Figure 8m). In contrast, devices containing 33% TFSI exhibited negligible retention (∼0.38 µA), highlighting the pivotal role of ion accumulation and retention in maintaining long‐term memory states. These results are analogous to the biological LTP phenomenon, where repeated presynaptic stimulation leads to increased neurotransmitter release and enhanced postsynaptic response efficiency. This finding suggests that neuromorphic devices are a fundamental technology that can extend beyond transient responses to include long‐term memory consolidation mechanisms. The gradual enhancement of electrical response under repeated mechanical stimulation demonstrates that the OEST‐based touch sensor (AOAN) is capable of performing tactile learning [347]. In practice, while conventional touch sensors failed to detect a light touch of approximately 2 kPa, such as the bounce of a ping‐pong ball, the AOAN exhibited improved sensitivity through repeated stimulation, ultimately enabling successful recognition (Figure 8n). This result highlights the synaptic functionality of the electronic skin, which not only detects stimuli but also adapts to and memorizes them.

This neural plasticity‐based signal amplification reflects an active interaction mechanism between external stimuli and electrical responses. In particular, the tribotronic active sensor (TAS) generates a triboelectric potential solely from external contact separation motions without any applied gate voltage, utilizing this potential as a gate bias to induce ion rearrangement and the formation of an EDL within the ion gel [348, 349, 350]. Upon contact, ions are randomly distributed throughout the gel, and the semiconductor channel maintains a flat energy band configuration. However, as the separation begins, negative charge induction drives ions toward the interface, resulting in hole accumulation, upward band bending, and a transition of the channel into an accumulation state, thereby increasing the current (Figure 8o) [351]. This change in transistor operation, visualized through the energy band diagram, indicates that the external mechanical stimulus directly modulates the device state. Such a mechanism provides the physical basis for the observed stimulus adaptation and enhanced sensitivity discussed earlier. Thus, TAS enables active modulation of the electronic device state using mechanical stimuli alone, and this characteristic can be extended as a core mechanism for implementing stimulus‐response coupling‐based neuromorphic associative learning. Such transistor‐based sensor structures contribute to continuous learning and memory formation in neuromorphic systems. A representative example is the emulation of Pavlov's classical conditioned reflex experiment as an associative learning scenario. In this scenario, a vibrational stimulus acts as a neutral stimulus analogous to a “bell sound,” while a pressure stimulus serves as an unconditioned stimulus corresponding to “food.” Initially, the vibrational stimulus alone does not elicit a postsynaptic current (EPSC) that exceeds the response threshold. However, through repeated paired training of the two stimuli, an association is formed, and subsequently, the vibrational stimulus alone can induce a response that exceeds the threshold (Figure 8p) [352, 353, 354]. This result demonstrates that the learning mechanisms of the nervous system can be effectively implemented at the device level, serving as a promising technological foundation for the development of biomimetic neural systems and electronic skin applications.

Neuromorphic devices integrated with ionic liquids represent an attempt to go beyond simple materials application, aiming to implement the complex and dynamic information processing mechanisms of the biological nervous system at the electronic device level. ILs offer the capability to flexibly and precisely mimic a sequence of biological processes, including sensory reception, ion‐based signal transduction, electrochemical plasticity, and self‐regulated learning. These features serve as a critical bridge that enables neuromorphic systems to evolve into architectures capable of sensing, memorizing, and responding to external stimuli in a manner functionally similar to the human nervous system. In the long term, IL‐based neuromorphic devices are establishing themselves as a foundational technology that translates biological principles into new forms of electronic cognition, decision‐making, and motor control. This approach not only pushes the boundaries of human–machine interfaces but also lays the groundwork for the development of more intelligent and intuitive electronic neural systems.

## 5 Conclusion

In summary, this review has explored the critical roles of ionic liquids in enabling advanced ion‐mediated communication between electronic and biological systems. By mimicking the dynamic and energy‐efficient ionic processes observed in physiological environments, ILs serve as artificial ions that emulate the behavior of native biological ions, thereby offering unique capabilities to bridge the ionic nature of biological signaling with the electronic framework of artificial devices. This review has outlined the ways in which ILs, owing to their chemical tunability and physical softness, support essential bioelectronic functions, including signal transduction, mechanical compliance, and synaptic modulation.

**TABLE 3:** Bulky and physical properties of biocompatible ILs.

Chemical name	Abbrev.	Melting point (*T_m_*) (°C)	Density (*ρ*) (g/cm^3^ at 20–25°C)	Viscosity (*η*) (mPa·s at 25°C)	Ionic conductivity (*σ*) (mS/cm at 25°C)	Electrochemical window (V)	Refs.
Choline lactate	[Ch][Lac]	< −40	1.14–1.20	250–450	0.5–2.0	∼2.3 (vs Ag/AgCl)	[367, 368]
Choline acetate	[Ch][Ace]	∼85	1.10–1.12	N/A	0.1–1.0	2.2–3.9	[369]
Choline propionate	[Ch][Prop]	< 25	∼1.10	∼ 300	∼1.5	1.8–2.5	[370]
Choline glycolate	[Ch][Glyc]	N/A	1.15–1.19	∼350	∼1.0	N/A	[371, 372]
Choline glycinate	[Ch][Gly]		1.14–1.15	182–300	67.7		
Choline alaninate	[Ch][Ala]		1.11–1.13	385–720	21.3		
Choline prolinate	[Ch][Pro]		1.12–1.14	9810–10 643	0.3–7.5		
Choline serinate	[Ch][Ser]		1.19–1.20	11 543–12 500	9.3		
Ethylammonium nitrate	EAN	12	1.21–1.26	36.5	20–25	2.5–3.0	[373]
Ethanoammonium acetate	2‐HEAA		1.10–1.11	390–450	2.0–2.3	∼2.1	

Building upon these foundation insights, the definitions, classifications, and molecular design strategies of ILs have been revisited. Key factors such as hydrophilicity/hydrophobicity, Hofmeister effects, and synthetic approaches are emphasized, as they strongly influence the interfacial behavior and compatibility of ILs with soft electronics systems. Furthermore, their functional roles at biointerfaces have been summarized in the context of electrochemical transduction, mechanical reinforcement of polymers, and stimuli‐responsive drug delivery, reinforcing their value as multifunctional enablers in bioelectronics. Despite the structural diversity and combinatorial potential of ILs, the types currently employed in bioelectronics remain limited, with their selection often relying on empirical criteria. Furthermore, quantitative analyses and detailed interfacial studies elucidating the chemical and physical mechanisms by which specific ILs influence device performance are still lacking. To overcome these limitations, it is imperative to establish a scientific understanding of the composition–structure–function relationships of ILs and to develop systematic design and analytical strategies that ensure their compatibility with the operating mechanisms of bioelectronic devices.

Ultimately, while each application, ranging from signal sensing and actuation to neuromorphic computing, demonstrates the versatility of ILs in specific functional roles, these are not isolated domains. Rather, they collectively illustrate how ILs act as a central unifying material platform that bridges diverse physiological processes through shared mechanisms of ion transport, electrochemical transduction, and mechanical adaptability. Across soft robotics, bioelectronic sensing, and neuromorphic interfaces, ILs facilitate localized yet coordinated control of charge dynamics and material–tissue interactions, enabling the construction of multifunctional systems that more closely resemble the complex, integrated nature of biological systems. Therefore, the future of IL‐based bioelectronics lies not only in the refinement of individual components, but also in the system‐level integration of sensing, stimulation, learning, and actuation functions that ILs are uniquely positioned to mediate. As this field advances, ILs will continue to serve as the molecular glue that enables convergence across domains, pushing bioelectronic technologies toward more adaptive, intelligent, and lifelike interfaces with the human body.

Despite these promising perspectives, several fundamental challenges must be overcome to translate IL‐based bioelectronics into commercially viable technologies. Many of these challenges arise from the inherently organic and mechanically soft nature of ionic liquids. While these characteristics provide clear advantages for biointerfacing, they can also compromise long‐term material and electrical stability. Further improvements in the performance of ILs are essential for realizing high‐performance bioelectronic devices. In particular, some IL‐based systems exhibit relatively low ionic conductivity and slower switching speeds compared to solid‐state dielectric materials, which may limit rapid signal transduction under high‐frequency operation. Such performance limitations introduce additional challenges for achieving device‐level reliability and reproducibility.

Among these issues, long‐term material stability remains a central concern. Although strategies that immobilize ionic liquids within elastomeric or polymer networks have been proposed to mitigate leakage and evaporation, additional studies are required to optimize diverse classes of ILs in combination with appropriate host matrices [217, 355, 356, 357, 358, 359]. At the same time, the hygroscopic nature of ILs presents both challenges and opportunities. While it necessitates careful materials design, it also enables emerging approaches such as ionic hydrogels that improve stability through controlled water uptake [157]. These stability considerations become even more critical for implantable applications. Preventing ILs leakage during prolonged exposure to complex biological environments is essential for maintaining device reliability and safety, thereby highlighting the importance of effective encapsulation strategies. Furthermore, regulatory approval for wearable and implantable applications requires a comprehensive evaluation of chronic biocompatibility, which remains limited for many existing IL systems. Continued development and validation of biocompatible bioionic liquids are, therefore, a key prerequisite for successful translation into practical applications. From the perspective of the device level, scalability and manufacturing constraints also represent significant barriers. The high viscosity and strong interfacial interactions of IL‐based gels complicate uniform printing, precise patterning, and reproducibility, thereby hindering large‐scale manufacturing. Addressing these challenges will require systematic, materials‐driven studies that establish clear relationships between IL composition, physicochemical properties, and device‐level performance. Building comprehensive libraries of IL‐device interactions through such efforts will be critical for enabling predictable, scalable bioelectronic platforms and for mitigating the intrinsic limitations of organic bioelectronic devices.

## Conflicts of Interest

The authors declare no conflicts of interest.

## Data Availability

The authors have nothing to report.
